# Non-coding RNAs underlie genetic predisposition to breast cancer

**DOI:** 10.1186/s13059-019-1876-z

**Published:** 2020-01-07

**Authors:** Mahdi Moradi Marjaneh, Jonathan Beesley, Tracy A. O’Mara, Pamela Mukhopadhyay, Lambros T. Koufariotis, Stephen Kazakoff, Nehal Hussein, Laura Fachal, Nenad Bartonicek, Kristine M. Hillman, Susanne Kaufmann, Haran Sivakumaran, Chanel E. Smart, Amy E. McCart Reed, Kaltin Ferguson, Jodi M. Saunus, Sunil R. Lakhani, Daniel R. Barnes, Antonis C. Antoniou, Marcel E. Dinger, Nicola Waddell, Douglas F. Easton, Alison M. Dunning, Georgia Chenevix-Trench, Stacey L. Edwards, Juliet D. French

**Affiliations:** 1 Cancer Division, QIMR Berghofer Medical Research Institute, Brisbane, Australia; 2 Current address: UK Dementia Research Institute, Imperial College London, London, UK; 3 Faculty of Medicine, The University of Queensland, Brisbane, Australia; 4 Centre for Cancer Genetic Epidemiology, Department of Oncology, University of Cambridge, Cambridge, UK; 5 Garvan Institute of Medical Research, Sydney, Australia; 6 UQ Centre for Clinical Research, Faculty of Medicine, The University of Queensland, Brisbane, Australia; 7 Pathology Queensland, The Royal Brisbane & Women’s Hospital, Herston, Brisbane, Australia; 8 Centre for Cancer Genetic Epidemiology, Department of Public Health and Primary Care, University of Cambridge, Cambridge, UK; 9 St Vincent’s Clinical School, University of New South Wales, Sydney, Australia

## Abstract

**Background:**

Genetic variants identified through genome-wide association studies (GWAS) are predominantly non-coding and typically attributed to altered regulatory elements such as enhancers and promoters. However, the contribution of non-coding RNAs to complex traits is not clear.

**Results:**

Using targeted RNA sequencing, we systematically annotated multi-exonic non-coding RNA (mencRNA) genes transcribed from 1.5-Mb intervals surrounding 139 breast cancer GWAS signals and assessed their contribution to breast cancer risk. We identify more than 4000 mencRNA genes and show their expression distinguishes normal breast tissue from tumors and different breast cancer subtypes. Importantly, breast cancer risk variants, identified through genetic fine-mapping, are significantly enriched in mencRNA exons, but not the promoters or introns. eQTL analyses identify mencRNAs whose expression is associated with risk variants. Furthermore, chromatin interaction data identify hundreds of mencRNA promoters that loop to regions that contain breast cancer risk variants.

**Conclusions:**

We have compiled the largest catalog of breast cancer-associated mencRNAs to date and provide evidence that modulation of mencRNAs by GWAS variants may provide an alternative mechanism underlying complex traits.

## Introduction

The human genome is extensively transcribed. The majority of transcripts are long non-coding RNAs (lncRNAs), defined as > 200 base pairs in length and transcribed antisense, intronic or intergenic to protein-coding genes. RNA sequencing studies conducted on different tissues and cell types are continually identifying new lncRNAs, which indicates that comprehensive annotation of lncRNA genes is far from complete [1–6]. Recent studies, using targeted RNA sequencing (also called RNA CaptureSeq), have revealed tremendous complexity of human transcriptomes—predominantly driven by pervasive transcription, complex alternative splicing, cell type, and context-specific gene expression [7, 8]. However, to date, only a small number of lncRNAs have an assigned function and the exact proportion of non-coding transcripts that are functional is a subject of continued debate.

Genome-wide association studies (GWAS) in combination with fine-mapping have identified 196 independent signals associated with breast cancer risk (conditional *p* values < 1 × 10^−6^) [9]. Sixty-six signals confer a greater risk of estrogen receptor (ER)-positive tumors, 29 confer a greater risk of developing ER-negative tumors, and the remaining signals showed no statistically significant difference in their effects on ER subtypes [9]. Due to complex linkage disequilibrium (LD), genetic variants within a signal are frequently co-inherited making it difficult to pinpoint the variant driving the association. A recent study has defined the credible causal variants (CCVs) within each signal as those with *p* values within two orders of magnitude from the lead variant (7394 CCVs/196) [9]. At 28 signals, a single CCV was identified, and at 96 signals, the number of CCVs was ≤ 10. Similar to other trait-associated variants identified by GWAS, the majority of CCVs are located in non-coding genomic regions [1].

We have recently shown that CCVs are enriched in genomic features associated with regulatory activity in a range of breast-derived cell lines including sites of open chromatin, chromatin marks associated with promoter and enhancer activity (H3K4Me3, H3K4Me1, and H3K27Ac), and transcription factor binding sites [9, 10]. Capture Hi-C analyses have identified annotated gene promoters that frequently interact with these CCV-containing elements providing a list of candidate risk genes for these signals [10]. However, at 20 signals, we found no evidence of CCVs falling in regions marked by regulatory activity suggesting that some signals alter risk through alternative mechanisms [10]. Using RNA CaptureSeq, we annotated multi-exonic non-coding RNAs (mencRNAs) transcribed from genome intervals surrounding breast cancer risk signals in a range of mammary-derived tissue and cell lines. We provide evidence that non-coding RNAs represent an alternative mechanism by which CCVs alter breast cancer risk. This likely extends beyond breast cancer and may represent a common mechanism by which GWAS variants act in other complex traits and diseases.

## Results

### RNA CaptureSeq array design and capture

**Fig. 1:**
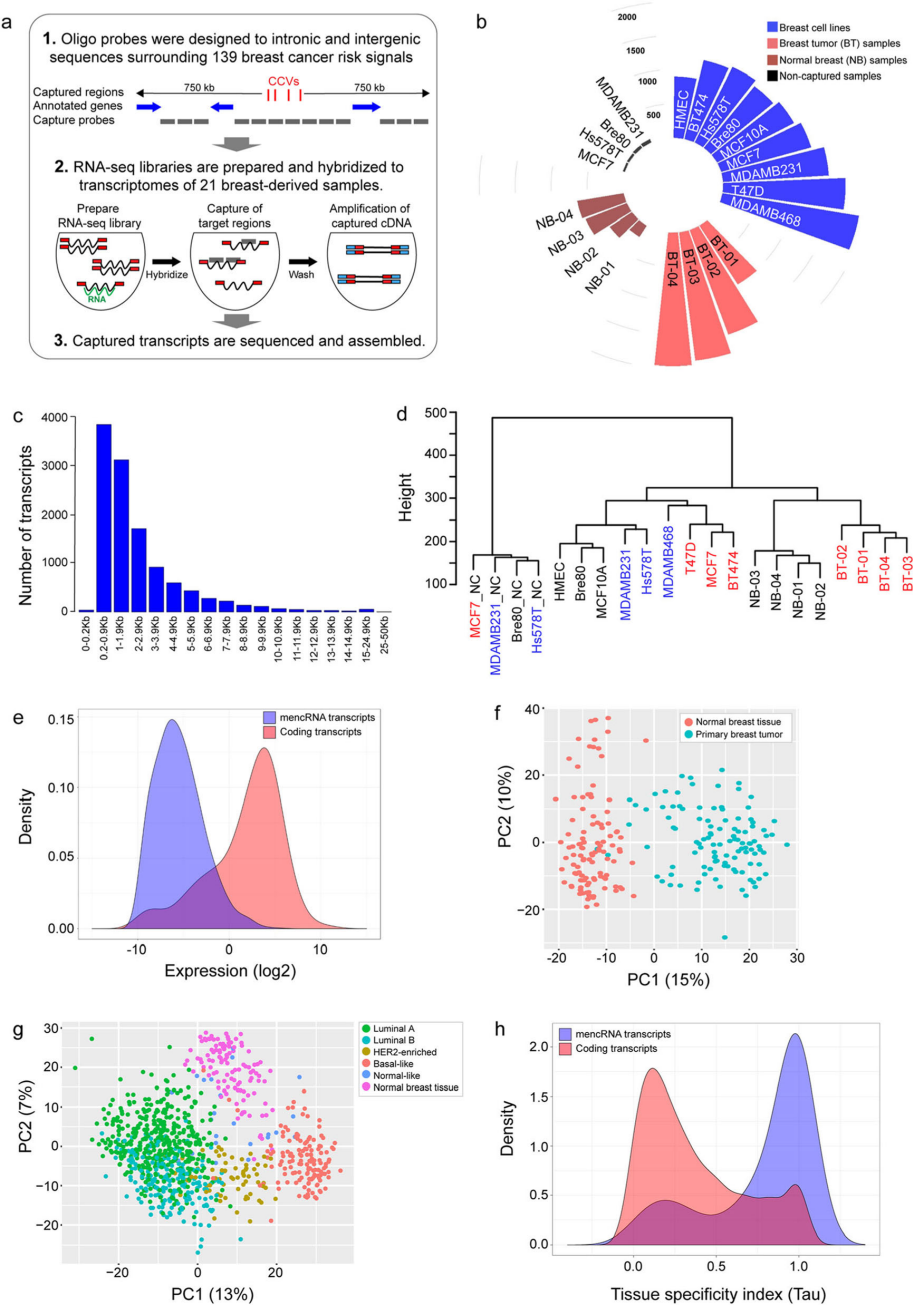
Identification of mencRNAs from breast cancer GWAS risk regions. **a** Schematic of the RNA CaptureSeq experimental design. Oligonucleotide probes were tiled across intronic and intergenic regions within 1.5-Mb intervals surrounding breast cancer risk regions (capturing ~ 138 Mb or 4.3% of the human genome). The probes were hybridized to cDNAs from breast-derived cell lines and tissues resulting in capture and enrichment of low abundance transcripts in target regions that were then sequenced. The sequencing reads were de novo assembled, mapped, and quantified. **b** The number of transcripts captured from each RNA CaptureSeq library. The libraries included nine breast-derived cell lines, four breast tumor (BT) samples, and four breast normal (NB) samples. Four non-captured libraries were also sequenced. **c** Distribution of mencRNA transcript length. Pooled captured transcripts from all libraries were binned based on their transcript lengths. **d** Hierarchical clustering of RNA CaptureSeq libraries based on mencRNA expression profiles. ER-positive breast cancer cell lines and tumors are shown in red, ER-negative breast cancer cell lines are shown in blue, and normal breast cell lines and tissues are shown in black. NC non-captured, NB normal breast, BT breast tumor. The *y*-axis of the dendrogram represents a distance measure between the clusters. **e** Expression distribution of captured mencRNA transcripts versus protein-coding transcripts. Multi-exonic captured transcripts with max. FPKM ≥ 0.5 were mapped in TCGA RNA-Seq data and their average expression across the TCGA tumors were compared to GENCODE protein-coding genes. The *y*-axis represents the frequency of transcripts with a given expression value represented as log2 (average FPKM) on the *x*-axis. **f** Principal component analysis (PCA) of captured transcripts in TCGA normal breast and matched tumor samples. Scaled, centred, and normalized expression of the captured transcripts were analyzed for the first (*x*-axis; PC1) and second (*y*-axis; PC2) principal components. Each dot represents expression profile of an individual sample. **g** PCA of the captured transcripts in different PAM50 breast cancer subtypes. **h** Comparison of tissue-specific expression of captured mencRNA versus protein-coding transcripts. Multi-exonic captured transcripts with max. FPKM ≥ 0.5 and GENCODE protein-coding genes were mapped in TCGA RNA-seq data for primary tumors from seven different cancer types. For each gene, tissue specificity index (Tau) was measured with 0 and 1, indicating broad and tissue-specific expression, respectively. The *y*-axis represents the frequency of transcripts with a given Tau value

To identify non-coding RNAs (ncRNAs) transcribed from breast cancer risk signals, we performed RNA CaptureSeq on 21 breast-derived samples including primary mammary epithelial cells from reduction mammoplasties, breast tumor samples, and normal mammary epithelial and breast cancer cell lines (Fig. 1a, b; Additional file 2: Tables S1 and S2). Oligonucleotide probes were designed to capture RNA transcripts produced from intronic and intergenic regions within 1.5-Mb intervals surrounding known breast cancer GWAS signals at the time of capture design (139/196 signals; Fig. 1a; Additional file 2: Table S3). Additional control sequences were added to the capture design to assess performance, including sequences corresponding to ERCC (External RNA Controls Consortium) spike-in transcripts and control housekeeping genes (Additional file 2: Table S4). In total, 138 Mb (4.3%) of the human genome was covered. RNA sequencing libraries were generated, hybridized against the capture probes, and sequenced. In addition, pre-captured RNA-seq libraries from four cell lines were sequenced to compare enrichment of the captured transcripts before and after hybridization. Based on the ERCC controls, the lower limit of detection (LLD), defined as the lowest molar amount of ERCC transcript detected in each library, was ~ 300 times lower across the four captured libraries compared to non-captured controls (Additional file 1: Figure S1). Moreover, the captured libraries showed a high enrichment of all control housekeeping genes included in the capture and no enrichment of off-target controls when compared to the non-captured libraries (*p* = 3.5 × 10^−10^; Additional file 1: Figure S2a).

### Annotation of mencRNAs at regions flanking 139 breast cancer risk signals

To discover novel ncRNA genes, sequencing reads from the 21 captured libraries were de novo assembled, mapped back to the genome, and quantified (Additional file 2: Table S2). ncRNA genes overlapping annotated coding sequences were excluded. Lowly expressed (maximum FPKM across the samples < 0.5) and single-exon genes were also removed to avoid artifacts derived from genomic DNA and transcriptional noise [7]. We identified 4020 mencRNA genes (FPKM ≥ 0.5), 2766 of which are novel (defined as not overlapping with lncRNAs reported by GENCODE v.19 [11], FANTOM-CAT [12], or NONCODE [13]) (Additional file 2: Tables S5 and S6). mencRNA transcript lengths ranged from 143 to 35,678 bp with a median length of 1550 bp (Fig. 1c). The Coding Potential Calculator [14] predicted a high proportion of the transcripts (94.03%) as “non-coding,” significantly more than GENCODE lncRNAs (chi-square *p* = 2.5 × 10^−5^; Additional file 2: Table S7). In silico assessment of the mencRNA transcripts showed the vast majority of splice junctions contained canonical dinucleotides (99.0%), were identified by uniquely mapping reads (81.3%), and were not overlapping with repeat sequences (96.1%) (Additional file 1: Figure S2b-d). Furthermore, similar to other studies [2], we show a high prevalence of two-exon lncRNA transcripts (Additional file 1: Figure S2e). On average, the mencRNA transcripts showed ~ 5.5 times lower expression compared to GENCODE lncRNAs detected by our RNA CaptureSeq (*n* = 328; Additional file 1: Figure S2f). This is likely due to the targeted RNA sequencing approach, which enabled the identification of transcripts with lower expression levels. Hierarchical clustering of the mencRNA expression profiles clustered the samples according to their estrogen receptor status, whether they were derived from normal or tumor samples or from primary tissue or cell lines (Fig. 1d). Thirty mencRNAs containing CCVs were prioritized for RT-PCR validation which was then performed on the captured transcripts, binned into three groups of ten based on their level of expression. Ninety percent of transcripts with a FPKM ≥ 2 were validated by RT-PCR (9/10) compared to 80% of transcripts with 0.5 ≤ FPKM < 2 and 30% of transcripts with FPKM < 0.5 (Additional file 1: Figure S3a).

As exposure to estrogen is known to promote the development of breast cancer, we treated MCF7 breast cancer cells with 17beta-estradiol to determine if expression of any mencRNAs was estrogen-regulated (Additional file 1: Figure S3b). Nearly one third of the mencRNAs (*n* = 1189) were differentially expressed between MCF7 estrogen-treated and vehicle-treated control libraries (FDR < 0.01; Additional file 1: Figure S3c; Additional file 2: Table S8). We then quantified the expression of our captured transcripts in 111 normal breast samples and 1092 breast tumors using standard RNA-seq datasets from TCGA [15]. Approximately 75% of the mencRNAs were present at an expression level of FPKM ≥ 1 even though no capture step had been performed prior to sequencing (Additional file 2: Table S9). Consistent with other non-coding RNAs, our mencRNAs had on average 140-fold lower expression compared with GENCODE protein-coding genes (Fig. 1e). Principal component analyses (PCA) based on mencRNA expression distinguished normal breast tissue from matched breast tumor, PAM50 breast cancer subtypes, and ER-positive versus ER-negative breast tumors (Fig. 1f, g; Additional file 1: Figure S3d). We also analyzed the expression of the mencRNAs in six additional tumor types and show that the mencRNAs exhibit high tissue specificity compared to protein-coding genes (Fig. 1h). PCA analyses based on mencRNA expression also tightly clustered tumors based on tissue type (Additional file 1: Figure S3e).

### Enrichment of CCVs for breast cancer risk in mencRNA exons

**Fig. 2:**
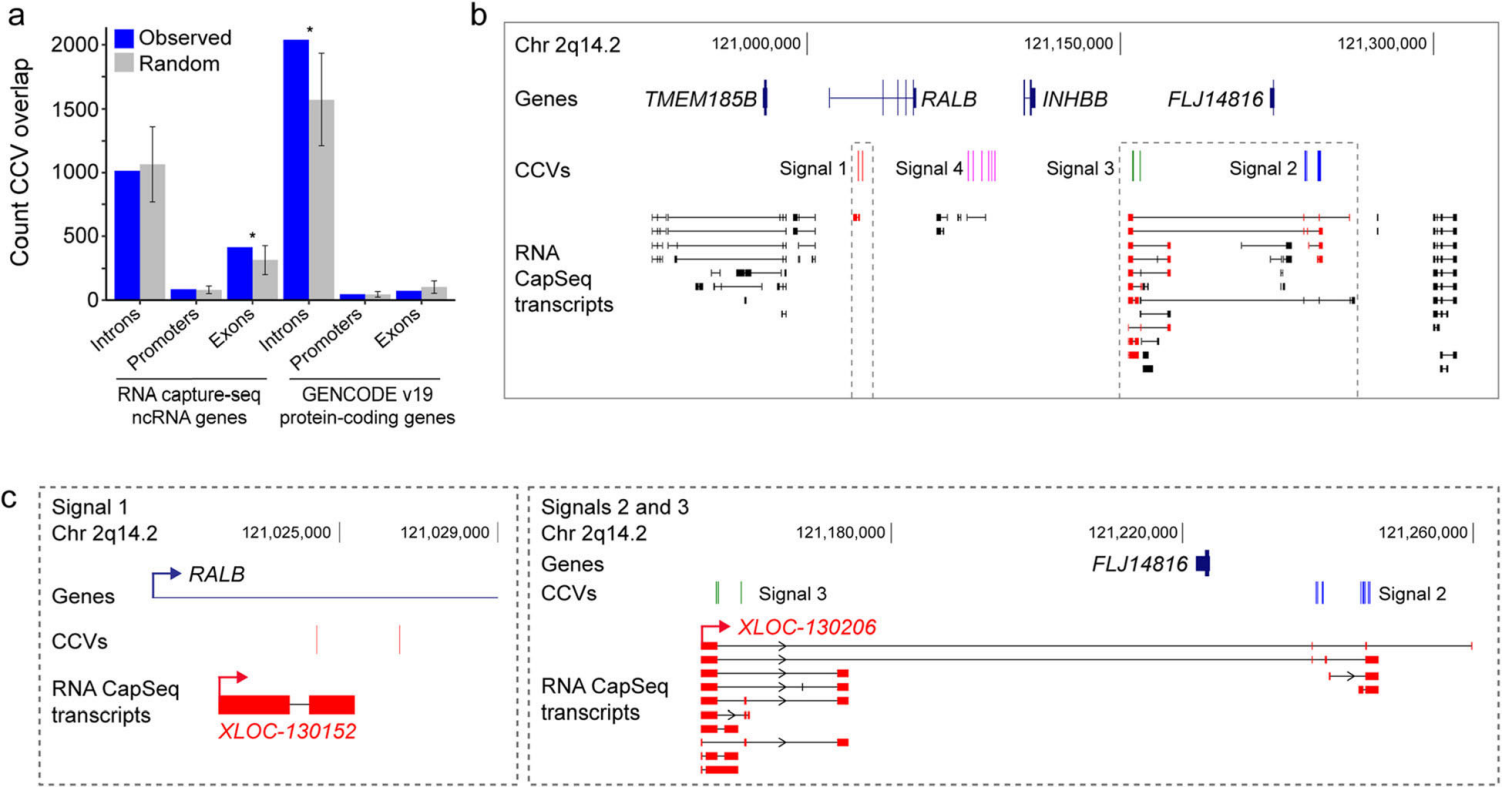
Enrichment of CCVs in mencRNA exons. **a** The overlap of CCVs with non-coding (ncRNA) and protein-coding transcript features. The number of CCVs directly overlapping a feature is shown in blue, and gray bars show the expected values based on overlap with 10^5^ randomly generated interval sets. Error bars show the 95% confidence intervals of the mean. The significance of the enrichment is expressed as *p* values, calculated by dividing the number of random samples showing equal or greater overlap than the observed by the total number of permutations (*p < 0.05). **b** WashU genome browser showing annotated GENCODE genes (blue), and mencRNAs (black) within the 2q14.2 risk region. The *XLOC-130152* and *XLOC-130206* mencRNAs are highlighted in red. Risk signals 1–4 are numbered and the CCVs within each signal shown as colored vertical lines. The dashed gray outlines highlight the CCVs and relevant mencRNAs. **c** Zoomed in view of signals 1–3 CCVs, *XLOC-130152* and *XLOC-130206*

Several studies have shown that most genetic variants identified through GWAS are non-coding and enriched in cell type-specific enhancers relevant to the GWAS trait [9, 16]. However, the contribution of non-coding RNAs to complex traits is not clear. We assessed the mechanisms underlying CCVs and mencRNAs and showed enrichment of CCVs in the exons, but not the promoters or introns of the mencRNAs (Fig. 2a). Conversely, CCVs were depleted in the exons of protein-coding genes and enriched in the introns. In total, 119 mencRNA genes at 69/139 signals contained a CCV (417 in total; Additional file 2: Tables S10 and S11) within a mencRNA exon. One example is the 2q14.2 risk region, where CCVs in 3 of the 4 independent risk signals fell within mencRNA exons (Fig. 2b, c). Of note, CCVs within signals 2 and 3 fell in different exons of the same mencRNA, *XLOC-130206* (Fig. 2c).

**Fig. 3:**
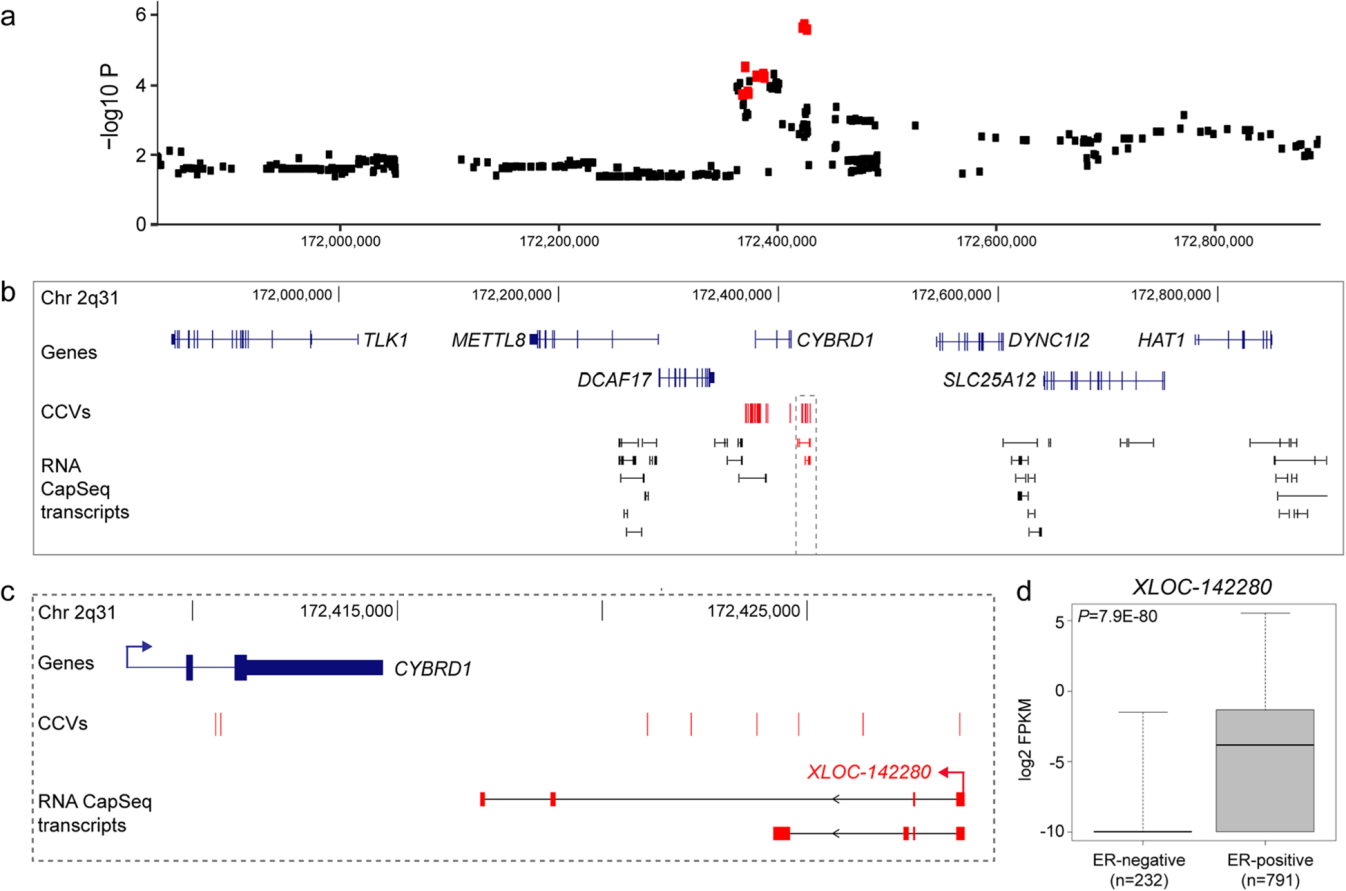
mencRNAs with eVariants in an exon. **a** Regional *XLOC-142280* eQTL association plot. Red dots indicate CCVs within the region. **b** WashU genome browser showing annotated GENCODE genes (blue) and mencRNAs (black) within the 2q31.1 risk region. The *XLOC-142280* mencRNA is highlighted in red. CCVs are shown as red colored vertical lines. The dashed gray outline highlights the *XLOC-142280* mencRNA. **c** Zoomed in view of CCVs and *XLOC-142280.* **d** Box plot showing the expression of *XLOC-142280* in ER-negative versus ER-positive breast tumor samples from TCGA RNA-seq data

Given the enrichment of CCVs in mencRNA exons, we hypothesized that CCVs could affect mencRNAs by modulating RNA stability. We therefore performed an expression quantitative trait loci (eQTL) study using the breast tumor TCGA datasets to identify genetic variations associated with mencRNA expression. We identified 800 mencRNAs which were eQTLs (FDR < 0.05), nine of these eQTLs overlapped with breast cancer signals, based on the *p* value for the top eQTL SNP being within two orders of magnitude of the eQTL *p* value for a CCV. We further examined the colocalization of the eQTL and breast cancer signals and show that seven of the nine eQTLs colocalize (Additional file 1: Figure S4; Additional file 2: Tables S12 and S13). Of these, three signals have at least one eQTL variant (eVariant) within a mencRNA exon (Additional file 2: Tables S12 and S13). For example, the eVariants at 2q31.1 (FDR = 0.002) fell within an exon of a mencRNA called *XLOC-142280* with the risk alleles associated with reduced expression (Fig. 3a–c; Additional file 1: Figure S5a; Additional file 2: Tables S12 and S13). Notably, eQTL studies in multiple normal and breast tumor cohorts did not find an association between CCVs at this signal and any annotated protein-coding genes (at *p* < 5 × 10^−4^), suggesting that *XLOC-142280* is a likely target gene [17]. Using TCGA RNA-seq data, we show that *XLOC-142280* is predominantly expressed in ER-positive breast cancers (Fig. 3d). Given that CCVs at this region are exclusively linked with ER-positive breast cancer, this association could be explained by the restricted breast cancer subtype expression of *XLOC-142280* [9].

### Evidence for distal CCVs modulating mencRNAs

**Fig. 4:**
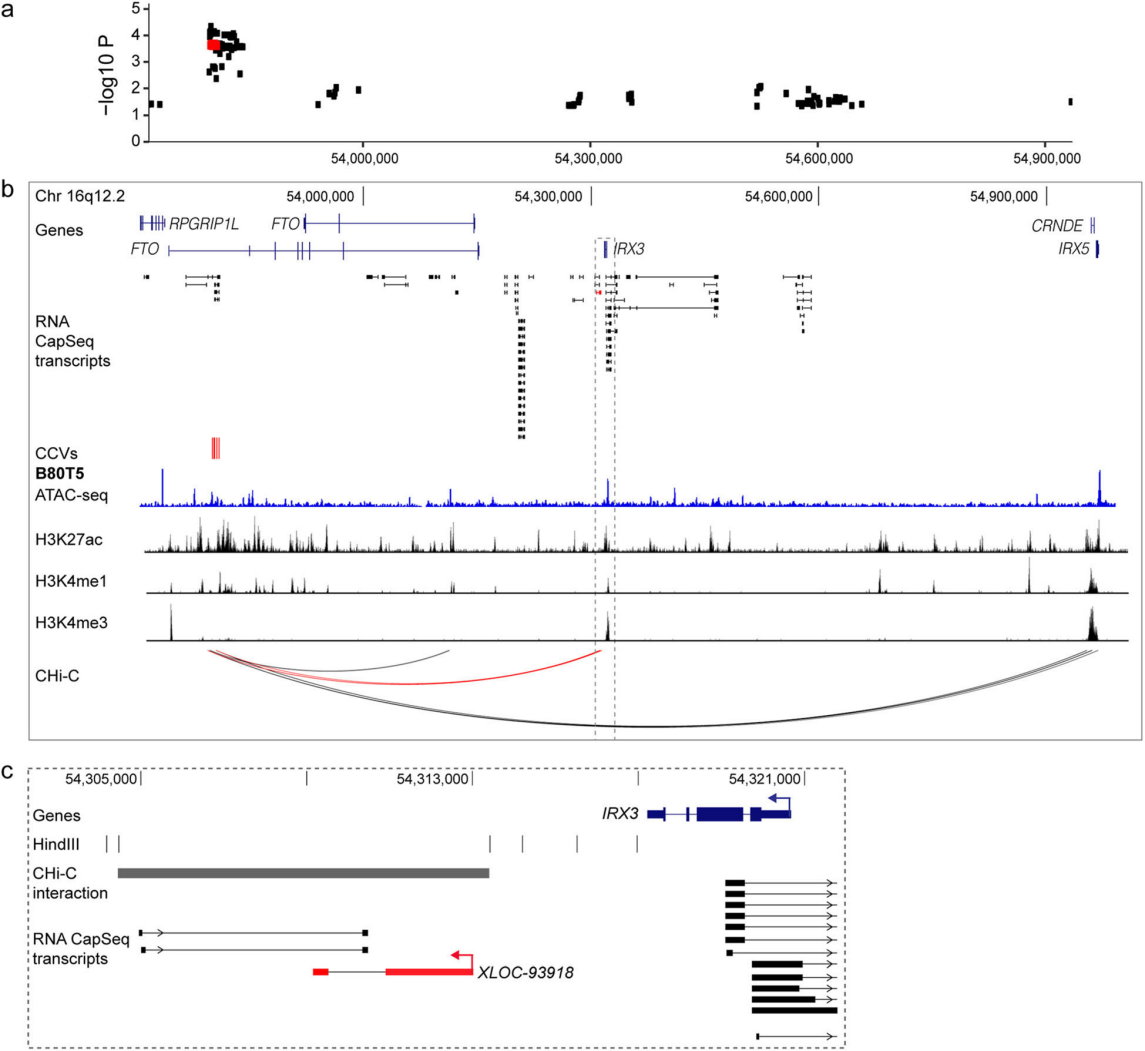
mencRNAs linked to distal CCVs at 16q12. **a** Regional *XLOC-93918* eQTL association plot. Red dots indicate CCVs within the region. **b** WashU genome browser showing annotated GENCODE genes (blue) and mencRNAs (black) within the 16q12.2 risk region. The *XLOC-93918* mencRNA is highlighted in red. CCVs are shown as red colored vertical lines. The ATAC-seq data is shown as a blue histogram, histone modification ChIP-seq data is shown as black histograms, and CHi-C chromatin interactions are shown as arcs from the B80T5 breast cell line. Red arcs depict chromatin looping between CCVs and the *XLOC-93918* promoter region. **c** Zoomed in view of CHi-C interaction and *XLOC-93918*

For 4/7 of the eQTLs that colocalized with breast cancer risk signals, there were no eVariants in mencRNA exons. Therefore, we speculated that CCVs may also act distally through regulatory elements that interact with mencRNA promoters. Using Capture Hi-C data from breast cells [10], we identified 770 mencRNA promoters (defined as ± 500 bp from the transcription start site) that looped to a region containing a CCV (Additional file 2: Tables S11 and S14). For example, at 16q12.2, CCV rs11642015 is an eVariant (*p* < 5 × 10^−4^) for the mencRNA *XLOC-093918*, with the risk haplotype associated with increased *XLOC-093918* levels (Fig. 4a; Additional file 1: Figure S5b; Additional file 2: Tables S12 and S13). CCV rs11642015 falls within a region of open chromatin marked by H3K27ac and H3K4me1, consistent with a putative enhancer element and contacts *XLOC-093918* in B80T5 normal breast cells and ER-positive and ER-negative breast cancer cell lines (Fig. 4b, c; Additional file 1: Figure S5c). The CCV also contacts the *IRX5/CRNDE* bidirectional promoter; however, no eQTL for *IRX5* or *CRNDE* or any other gene was detected in either normal or tumor tissues (at *p* < 5 × 10^−4^) [17].

**Fig. 5:**
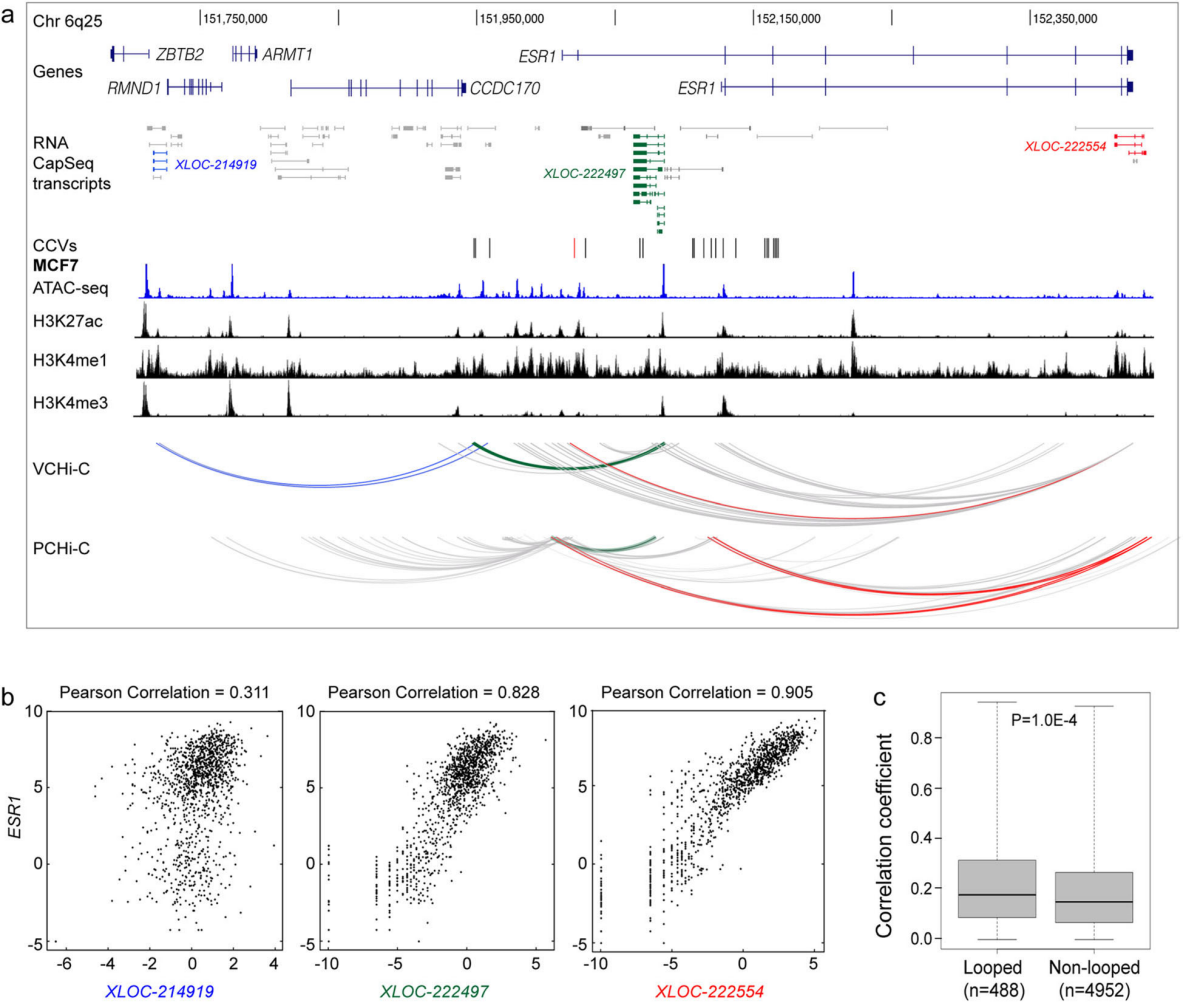
mencRNAs linked to distal CCVs at 6q25. **a** WashU genome browser showing annotated GENCODE genes (blue) and mencRNAs (black) within the 6q25 risk region. mencRNAs whose promoters participate in chromatin interactions with CCVs are highlighted in blue, green, and red. CCVs are shown as colored vertical lines. The ATAC-seq data is shown as a blue histogram, histone modification ChIP-seq data is shown as black histograms, and CHi-C chromatin interactions are shown as arcs from the MCF7 breast cancer cell line. Colored arcs depict chromatin looping between CCVs and color-matched mencRNA promoter regions. **b** Correlation between expression of the three captured transcripts and *ESR1* in the TCGA cohort. Each dot in the scatterplots represents a breast cancer individual with gene expression values being plotted as log2 (FPKM) on the *x*- and *y*-axes. **c** Boxplot showing absolute correlation coefficient values compared between looped and non-looped pairs of mencRNAs and nearby protein-coding genes (within 1 Mb up- and downstream)

In another example, we show that promoters of three mencRNAs (*XLOC-214919*, *XLOC-222497*, and *XLOC-222554*) located at the 6q25/*ESR1* risk region participate in chromatin looping with regions containing CCVs (Fig. 5a). Interestingly, we also observed looping between the *ESR1* promoter and both *XLOC-222497* and *XLOC-222554* (Fig. 5a) and the expression of both mencRNAs was highly correlated with *ESR1* (Fig. 5b), suggesting that these genes are co-regulated or are themselves regulatory mencRNAs that mediate *cis*-regulation of *ESR1*. Given the high correlation between the mencRNA-mRNA pairs at 6q25, which are connected by chromatin loops, we investigated whether mencRNA-mRNA pairs connected by loops across our captured regions were more highly correlated than mencRNA-mRNA pairs not connected. Although the effect size is marginal, we found that the expression of mencRNA-mRNA pairs that are physically connected through chromatin looping are significantly more correlated than a random set of mencRNA-mRNA pairs (Fig. 5c).

### Evidence for mencRNAs being the target gene of more than one signal

**Fig. 6:**
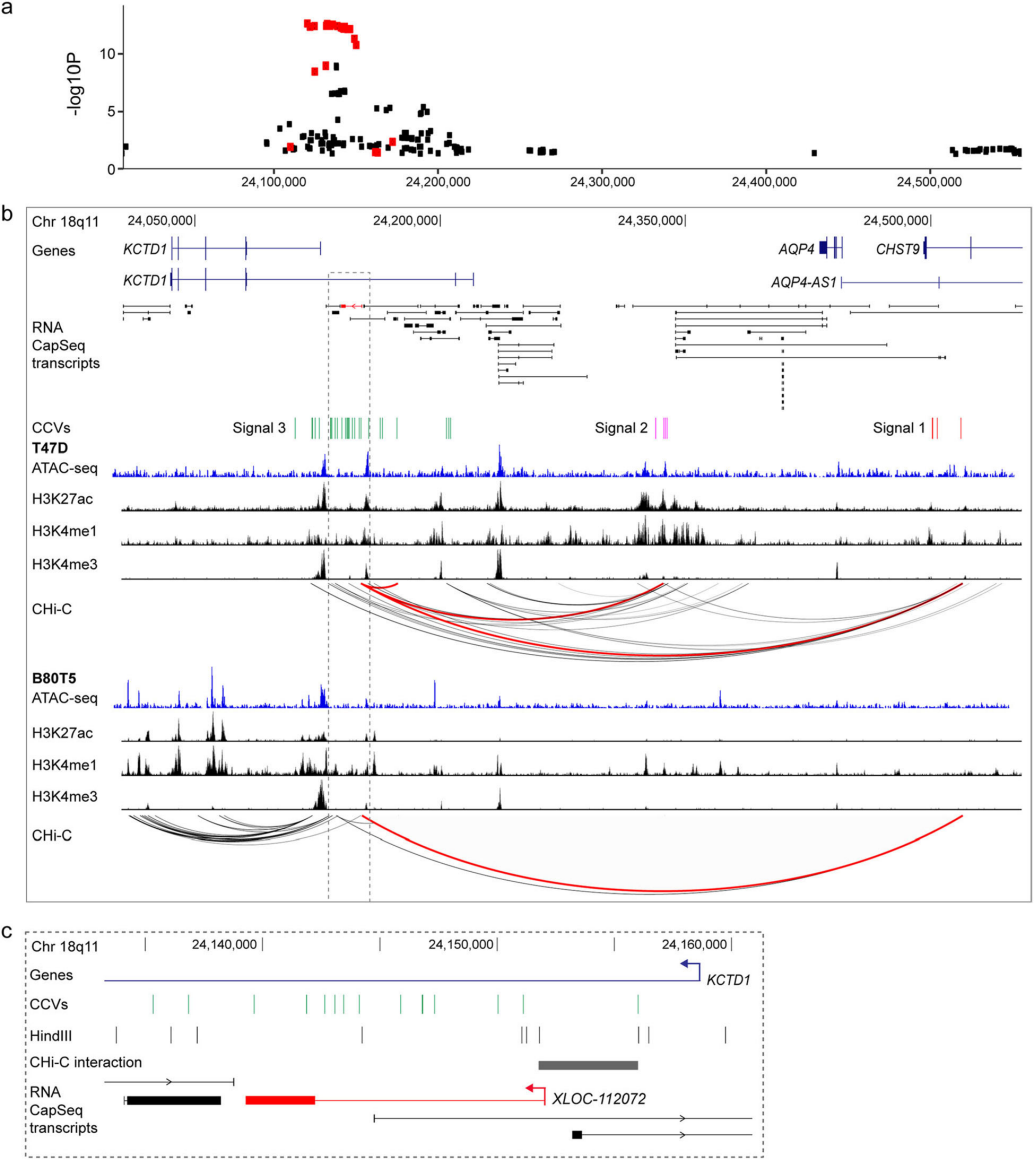
mencRNAs targeted by multiple risk signals. **a** Regional *XLOC-112072* eQTL association plot. Red dots indicate signal 3 CCVs. **b** WashU genome browser showing annotated GENCODE genes (blue) and mencRNAs (black) within the 18q11 risk region. The *XLOC-112072* mencRNA is highlighted in red. Risk signals 1–3 are numbered and the CCVs within each signal shown as colored vertical lines. The ATAC-seq data are shown as blue histograms, histone modification ChIP-seq data is shown as black histograms, and CHi-C chromatin interactions are shown as arcs from T47D and B80T5 breast cell lines. Red arcs depict chromatin looping between CCVs and the *XLOC-112072* promoter region. **c** Zoomed in view of CCVs, CHi-C interaction, and *XLOC-9112072*

Recent fine-scale mapping of breast cancer GWAS risk regions have reported multiple independent risk signals, some of which target the same protein-coding gene [9, 10]. We therefore sought to identify mencRNAs that are targeted by more than one risk signal. We identified 222 mencRNA genes in which two or more independent CCVs fell either (i) within the exon of the mencRNA, (ii) within the promoter region of the mencRNA, or (iii) in a region that interacts through long-range chromatin interactions with the promoter of the mencRNA (Additional file 2: Table S14). For example, at 18q11.2, we identified an eQTL variant for *XLOC-112072* (FDR = 1.95 × 10^−10^) that overlaps signal 3 CCVs (Fig. 6a–c; Additional file 1: Figure S5d), two of which fall in the mencRNA exon. In addition, signals 1 and 2 loop to the promoter of *XLOC-112072* in T47D breast cancer cells (Fig. 6b). Interestingly, only signal 1 interacts with the *XLOC-112072* promoter in B80T5 normal breast cells, suggesting some level of cell-type specificity (Fig. 6b). Notably, no other protein-coding eQTLs were identified for signals 1, 2, or 3 (*p* < 5 × 10^−4^), supporting a role for this mencRNA as one of the likely target genes at this breast cancer risk signal.

## Discussion

To date, the major focus of GWAS follow-up studies has been the impact of regulatory variants on the expression of protein-coding genes. In many cases, genetic variants with stronger genetic associations with the trait of interest are dismissed as causal because they do not fall in regions marked for promoter or enhancer activity. Using a combination of targeted RNA sequencing and de novo transcript assembly, we annotated > 4000 mencRNA genes expressed from genomic intervals surrounding 139 breast cancer GWAS signals. We identify 844 mencRNAs as candidate breast cancer risk genes based on CCVs that fall in either (1) mencRNA exons, (2) mencRNA promoters, or (3) regions that interact with the mencRNA promoters through long-range chromatin interactions. We summarize the evidence for mencRNA involvement at breast cancer risk signals (Additional file 2: Table S11), which will facilitate future lab-based functional studies. Collectively, these results suggest that modulation of mencRNAs may represent an alternative mechanism by which CCVs contribute to risk.

Our eQTL analyses identified nine mencRNA eQTLs which colocalize with breast cancer risk signals. Of these, five were the only detectable eQTL for the signal providing evidence that these mencRNAs are at least one of the likely candidate target genes at the respective signal. Three of the seven mencRNAs had an eVariant within the mencRNA exon. However, there were other genetic variants in LD which were more distal (CCVs in the same signal), making it difficult to determine if the exonic eVariant is affecting the mencRNA stability and driving the association. For example, the CCVs within the same signal that lie outside of mencRNA exons could potentially effect mencRNA expression through altered *cis*-regulatory elements (e.g., mencRNA promoters or enhancers). Consistent with this, for 4/7 mencRNA eQTLs that overlap breast cancer risk signals, the eVariants fall outside the mencRNA exons suggesting that, in these cases, altered distal regulation is the more likely mechanism. We have already demonstrated this regulatory mechanism at the 11q13 breast cancer risk region [18]. CCVs at 11q13 fall within an estrogen-regulated enhancer of two lncRNAs called *CUPID1* and *CUPID2.* In heterozygous breast cancer cell lines, we showed allele-specific chromatin looping between the enhancer and the *CUPID1*/*2* bidirectional promoter suggesting CCVs reduce their expression by inhibiting chromatin looping [18]. *CUPID1*/*2* play a role in modulating pathway choice for the repair of double-stranded DNA breaks by promoting homologous recombination-based repair [18], providing a plausible mechanism by which CCVs alter breast cancer risk. In another example, prostate cancer risk variants at 8q24 increase the activity of an enhancer for the *PCAT1* lncRNA. *PCAT1* interacts with the androgen receptor and lysine-specific demethylase to promote prostate cell growth providing a mechanism by which genetic variants increase risk of prostate cancer [19].

We show that CCVs are enriched in the exons but not the introns or promoters of mencRNAs, suggesting that genetic variants may alter mencRNA structure and/or function. lncRNAs can act as protein scaffolds; therefore it is possible that a genetic variant could alter the binding of proteins to mencRNAs. For example, *LINP1* promotes the repair of DNA breaks by acting as a scaffold for proteins involved in non-homologous end joining (NHEJ) [20]. The 2q12.1 region associated with celiac disease provides an example of genetic variants affecting lncRNA function at a GWAS locus. This study showed that risk-associated variants repress the expression of inflammatory genes by altering the secondary structure of a lncRNA called *Lnc13* and ultimately the binding of hnRNPD to *Lnc13* [21]. At some breast cancer risk signals where no eQTL association was found, we speculate that CCVs may be functional without affecting gene expression. This is certainly the case for somatic mutations in two lncRNAs, *MALAT1* and *NEAT1*, where levels of these lncRNAs are not significantly altered in mutated vs non-mutated samples [22].

CCVs are also enriched in genomic features associated with *cis*-regulatory DNA elements including sites of open chromatin, chromatin marks associated with promoter and enhancer activity, and transcription factor binding sites [9, 10]. Given that lncRNAs often arise from enhancer elements [23–25], it is possible that some CCV-containing enhancers fall within mencRNAs. Notably, we did not observe enrichment of CCVs in the introns or promoter regions of mencRNAs, suggesting that alteration of the lncRNAs transcribed from enhancers may have a functional effect on the enhancer activity. Several lncRNAs are required for promoting chromatin looping between enhancers and promoters [26–28]. Therefore, it is possible that genetic variants within the lncRNA could affect this process. Further work will be required to determine whether the CCVs that overlap mencRNAs and enhancers in this study act through the DNA element, for example, by altering transcription factor binding, or whether the variant affects the RNA transcript itself.

## Conclusions

In summary, we have compiled the largest catalog of breast cancer-associated mencRNAs to date and provide evidence that modulation of mencRNAs by GWAS variants may provide an alternative mechanism underlying complex traits. These findings have broad implications for interpreting findings from GWAS and suggest that comprehensive annotation of ncRNAs expressed in relevant cell types around GWAS signals will be important for functional follow-up studies. We do not yet know what proportion of mencRNAs identified in this study are functional. However, even if a small percentage have evolved functions, this resource may contain hundreds of new genes that hold the key to understanding breast cancer etiology. lncRNAs display remarkable cell and tissue-specific expression making them excellent candidates for therapeutic targets. Understanding the function of these lncRNAs therefore holds great potential for the development of new breast cancer therapies.

## Methods

### Availability of data and materials

Processed RNA sequencing and chromatin interaction data can be visualized at the Washington Epigenome Browser via https://bit.ly/2E1EpmC. Processed RNA sequencing data is available from https://osf.io/ew86n/?view_only=8d2c5c17dff54fb98daa3cfe51d2d473. Raw sequencing data has been deposited at the European Genome-phenome Archive (EGA) which is hosted at the EBI and the CRG, under the accession number EGAS00001003353. The custom scripts used in the study are available from https://github.com/MahdiMoradiMarjaneh/ncRNAs_BRCA_GenomeBiol2019. All datasets and software used are listed in Additional file 2: Table S16 [10, 14, 29–36].

### Breast cell lines

ER-positive breast cancer cell lines MCF7, T47D, and BT474 were grown in RPMI medium with 10% (vol/vol) fetal calf serum (FCS), 1 mM sodium pyruvate, 10 μg/mL insulin, and 1% (vol/vol) antibiotics. ER-negative breast cancer cell lines MDA-MB-231, MDA-MB-468, and Hs578T were grown in RPMI medium with 10% (vol/vol) FCS and 1% (vol/vol) antibiotics. The normal breast epithelial cell line MCF10A was grown in DMEM/F12 medium with 5% (vol/vol) horse serum, 10 μg/ml insulin, 0.5 μg/ml hydrocortisone, 20 ng/ml epidermal growth factor, 100 ng/ml cholera toxin, and 1% (vol/vol) antibiotics. The normal breast epithelial cell line B80-hTERT1 (provided by Roger Reddel, CMRI, Australia) was grown in a 1:1 mixture of MCDB 170 and RPMI 1640 media with 10% (vol/vol) FCS and 1% (vol/vol) antibiotics. Primary human mammary epithelial cells (HMECs) were grown in basal medium (MEBM, Lonza) supplemented with SingleQuots (MEGM BulletKit, Lonza). Cell lines were maintained under standard conditions (37 °C, 5% CO_2_), tested for *Mycoplasma*, and profiled for short tandem repeats.

### Human samples

Normal breast samples were derived from reduction mammoplasty from four donors, and organoids were obtained according to Johnson et al. [37].

### RNA CaptureSeq array design

Oligonucleotide probes were designed to capture non-coding sequences within 1.5-Mb intervals surrounding breast cancer GWAS signals using Roche NimbleGen’s capture design algorithm. To evaluate performance of the RNA CaptureSeq, control sequences were added to the design including 92 ERCC spike-in transcripts and 9 housekeeping genes covering a range of expression levels across breast tissues (*ABCB1*, *GATA1*, *GUSB*, *HMBS*, *HPRT1*, *NLK*, *RUNX2*, *TBP*, and *TFRC*; Additional file 2: Table S4). In total, 85.8% of the target bases were captured providing a total capture size of 137.7 Mb (4.3% of the human genome). The vast majority (~ 92%) of capture probes were unique (matched to only one genomic sequence). To increase the coverage of the remaining targets, a small proportion had multi-sequence homology.

### RNA CaptureSeq library preparation and capture sequencing

RNA was extracted from the primary mammary epithelial cells and normal mammary epithelial and breast cancer cell lines using the RNeasy Plus Mini Kit (Qiagen). Five micrograms of total RNA was rRNA depleted using the Ribo-Zero Gold rRNA removal kit according to the manufacturers’ instructions (Illumina). RNA was extracted from the breast tumor samples using TRIzol (Thermo Fisher Scientific), then DNase-treated with Turbo DNase (Life Technologies). ERCC RNA spike-in control mix 1 or 2 (Life Technologies) were added to ribodepleted RNA (final dilution of 1/50) or to 200 ng of total RNA (from breast tumors; final dilution of 1/1250). RNA-seq libraries were generated using the KAPA stranded RNA-seq Library Preparation Kit (Roche) and 12 cycles of pre-capture LM-PCR. Multiplex library pools were created by mixing equal amounts of four pre-capture libraries and capture hybridization performed on 1 μg of the pooled library. Capture hybridizations were performed using the SeqCap RNA Enrichment System (NimbleGen) according to the manufacturer’s instructions. Post-capture LM-PCR was performed for 14 cycles. One library pool (representing four original libraries) was sequenced per lane on the Illumina HiSeq2500 platform (Kinghorn Centre for Clinical Genomics, Sydney, Australia).

### Assessment of RNA CaptureSeq performance

To evaluate fold enrichment, sensitivity, and specificity of the RNA CaptureSeq, we added control sequences to the capture design including ERCC spike-in transcripts and housekeeping genes. Before hybridization of the libraries to the probes, we made technical replicates from four pre-capture libraries (B80-hTERT1, Hs578T, MCF7, and MDA-MB-231) and sequenced. Sequencing data from these non-captured and the corresponding captured libraries were used to evaluate the RNA CaptureSeq performance. Adapter trimming and quality control of the sequencing reads were performed with Trim Galore version 0.3.7. The reads were then mapped against hg19 reference genome appended with 92 ERCC spike-in transcripts using STAR version 2.4.2a. RSEM version 1.2.25 was used for transcript quantification. Expression data for the ERCC spike-in transcripts were used to assess sensitivity and fold enrichment of the RNA CaptureSeq. A dose-response plot for each library was generated by plotting normalized expression (log2 (FPKM)) of the ERCC transcripts against their known molar concentration added by a linear regression line of best-fit. To assess the sensitivity of the platform, we obtained a lower limit of detection (LLD) from each plot (defined as the lowest concentration of the ERCC transcripts present in each library that yields a detectable expression with the detection threshold FPKM of 0.5) and compared between captured and non-captured libraries. Upper limits of detection were also determined to assess probe saturation. Fold enrichment of the platform was calculated using linear regression equations of the captured and non-captured libraries. We evaluated off-target capture as a measure of specificity of the RNA CaptureSeq. Seven housekeeping genes included in the design and expressed in the samples (*GUSB*, *HMBS*, *HPRT1*, *NLK*, *RUNX2*, *TBP*, *TFRC*) were tagged as “targeted” and, for each, the nearest protein-coding gene not included in the design (*VKORC1L1*, *H2AFX*, *PHF6*, *TMEM97*, *SUPT3H*, *PSMB1*, and *ZDHHC19*) was selected and tagged as “non-targeted.” For each gene, expression fold change between the captured and non-captured libraries was measured and log-transformed values were compared between the targeted and non-targeted genes using a paired Student’s *t* test.

### De novo assembly and transcript characterization

As described previously [7], for each library, the sequencing reads were trimmed with Trim Galore version 0.3.7, assembled with Trinity version 2.2.0 [29], and mapped back to the hg19 reference genome with GMAP version 2015-11-20 [30]. The transcripts from all libraries were then merged together using the Cuffmerge function of Cufflinks version 2.2.1 [31]. The sequencing reads were then mapped against the merged assembly using STAR version 2.4.2a [32] and quantified using RSEM version 1.2.25 [33]. Single-exon genes, those not overlapping breast cancer GWAS regions, and those overlapping protein-coding sequences were removed. Moreover, we excluded lowly expressed genes (maximum FPKM across all libraries < 0.5) and isoforms with very low expression (maximum FPKM across all libraries < 0.01). The identified mencRNAs were assessed for coding potential by Coding Potential Calculator [14]. Splice junctions were annotated and characterized using STAR version 2.4.2a. We clustered the samples based on expression profiles of the mencRNAs using the “hclust” function from the R “Stats” package. EdgeR Bioconductor package [34] was used to identify mencRNAs differentially expressed between MCF7 estrogen-treated and vehicle-treated control libraries.

### Annotation of the captured transcripts in TCGA cohort

The captured transcripts were filtered for multi-exon genes and those overlapping with GWAS regions, then merged with the GENCODE comprehensive gene annotation (release 19) to generate a custom reference annotation file. In the case of overlapping genes, if the RNA CaptureSeq gene was lowly expressed (maximum FPKM across all libraries < 0.5), it would be removed and otherwise the GENCODE gene would be excluded. TCGA RNA-seq data for breast invasive carcinoma cohort (1226 samples), cervical squamous cell carcinoma (*n* = 309), esophageal carcinoma (*n* = 165), mesothelioma (*n* = 86), prostate adenocarcinoma (*n* = 552), skin cutaneous melanoma (*n* = 463), and uterine corpus endometrial carcinoma (*n* = 175) cohorts was obtained from Cancer Genomics Hub. Adaptor trimming of the sequencing reads was performed using Cutadapt version 1.11. Using the custom reference annotation file, the reads were then mapped with STAR version 2.5.2a and quantified with RSEM version 1.2.30 [32, 33]. Since TCGA RNA-seq data is not strand-specific, we excluded the captured transcripts which overlap (≥ 1 bp) with exons of protein-coding genes on the opposite strand in order to prevent bias when assessing expression levels. For differential expression analysis, genes with low expression (less than five counts per million in less than five samples) were excluded. The remaining expression data were imported into edgeR Bioconductor package [34] and normalized for library size and RNA composition. Differential expression analysis was then performed using edgeR functions *glmFit* and *glmLRT*. We used the R function *prcomp* for principal component analysis (PCA). For co-expression analysis, genes with low expression (expression counts in less than 20% of samples) or low variability (median absolute deviation (MAD) across the samples < 1) were excluded. Pairwise correlations were then computed using the R *cor* function. Tissue specificity index (Tau) [38] was measured using log2-transformed expression data for primary tumor samples from the seven TCGA cancer cohorts.

### RT-PCR validation of the captured lncRNAs

We used RT-PCR to validate 30 captured lncRNAs, binned into three groups based on their level of detection in T47D breast cancer cells. mencRNAs expressed at FPKM > 2 were classified as high expressed, 0.5 ≤ FPKM < 2 as moderate expressed, and FPKM < 0.5 as low expressed. Five micrograms of total RNA was first reverse transcribed using SuperScript III (Life Technologies), and PCR was performed using MyTaq DNA polymerase according to the manufacturers’ instructions. Forty cycles were performed in order to identify low expressed transcripts. Forward primers and reverse primers were designed to different mencRNA exons to avoid amplification of genomic DNA. Primers are listed in Additional file 2: Table S15.

### CCV enrichment analyses

We first examined the overlap of CCVs and mencRNAs genes. Variants were intersected with mencRNA exons, introns, and promoter sequences (defined as 500 bp upstream of the transcription start site). Fold enrichment was calculated by dividing the proportion of CCVs that overlapped the annotations by the proportion of coverage of that annotation within the captured regions. The significance of the overlap was calculated by comparing it to the expected using a hypergeometric test using Bedtools fisher. To further analyze CCV overlap, positions of each annotation were permuted around circularized capture regions. This approach maintained the relative sizes and positions of annotations belonging to the same transcript. Overlap of CCVs was compared to the mean overlap for 10^5^ random permutations.

### mencRNA eQTL analyses

RNA-seq reads from TCGA breast invasive carcinoma cohort were mapped against mencRNAs and quantified as described above. For these analyses, we did not filter the mencRNAs based on RNA CaptureSeq expression. The expression data for tumors (*n* = 1112) were then analyzed for eQTL detection. Patient germline SNP genotypes (Affymetrix 6.0 arrays) were processed and imputed to the 1000 Genomes reference panel (October 2014) as previously described [17]. Tumor tissue copy number was estimated from the Affymetrix 6.0 arrays and called using the GISTIC2 algorithm [35]. Complete genotype, RNA-seq, and copy number data were available for 678 genetically European patients. The FPKM values were log2 transformed and quantile normalized. Genetic variants within the captured regions were extracted from the imputed genotype dataset. The associations between genetic variants and mencRNA expression in breast tumor tissue were evaluated using linear regression models by the MatrixEQTL program in R [36]. Tumor tissue was also adjusted for copy number variation, as previously described [39]. A false discovery rate of 5% was used to report eQTL results from breast tissue. We considered eQTLs to overlap breast cancer risk signals based on the *p* value for the top eQTL SNP being within two orders of magnitude of the eQTL *p* value for a CCV. Colocalization analysis of eQTL and breast cancer risk signals was performed as described by Liu et al. [40].

### ATAC-seq and Capture Hi-C

ATAC-seq and Capture Hi-C in breast cells were obtained from Beesley et al. [10].

## Supplementary information

**Additional file 1: Figure S1.** RNA CaptureSeq performance. **Figure S2.** Quality control and properties of captured transcripts. **Figure S3.** Validation of captured transcripts. **Figure S4.** Co-localisation of eQTL associations at breast cancer GWAS signals. **Figure S5.** eQTL analysis and chromatin interactions at breast cancer risk regions.

**Additional file 2: Table S1.** Breast derived samples on which RNA CaptureSeq was performed. **Table S2.** Expression (FPKM) of the mencRNAs across all RNA CaptureSeq samples. **Table S3.** Breast cancer risk signals and surrounding regions captured by RNA CaptureSeq. **Table S4.** Control sequences captured by RNA CaptureSeq. **Table S5.** MencRNAs with FPKM ≥0.5 identified by RNA CaptureSeq. **Table S6.** Novel mencRNAs with FPKM ≥0.5. Those overlapping with GENCODE, FANTOM-CAT, or NONCODE lncRNAs have been excluded. **Table S7.** Coding potential of the transcripts from mencRNAs with FPKM ≥0.5 assessed by coding potential calculator (CPC2). **Table S8.** MencRNAs differentially expressed between MCF7 estrogen-treated and control samples (FDR < 0.01). **Table S9.** MencRNAs expressed in TCGA with FPKM ≥1 in at least one sample. **Table S10.** Count of CCVs overlapping transcript features. **Table S11.** Breast cancer CCVs linked to genomic features. **Table S12.** MencRNA eQTLs. **Table S13.** Colocalized mencRNA eQTLs. **Table S14.** MencRNAs targeted by breast cancer risk signals. **Table S15.** Primers used for mencRNA validation. **Table S16.** Publically available genomic data and software.

**Additional file 3.** Review history.

## References

[1] Cabili MN, Trapnell C, Goff L, Koziol M, Tazon-Vega B, Regev A (2011). Integrative annotation of human large intergenic noncoding RNAs reveals global properties and specific subclasses. Genes Dev.

[2] Derrien T, Johnson R, Bussotti G, Tanzer A, Djebali S, Tilgner H (2012). The GENCODE v7 catalog of human long noncoding RNAs: analysis of their gene structure, evolution, and expression. Genome Res.

[3] Djebali S, Davis CA, Merkel A, Dobin A, Lassmann T, Mortazavi A (2012). Landscape of transcription in human cells. Nature..

[4] Sun M, Kraus WL (2015). From discovery to function: the expanding roles of long noncoding RNAs in physiology and disease. Endocr Rev.

[5] Ulitsky I, Bartel DP (2013). lincRNAs: genomics, evolution, and mechanisms. Cell..

[6] Volders PJ, Helsens K, Wang X, Menten B, Martens L, Gevaert K (2013). LNCipedia: a database for annotated human lncRNA transcript sequences and structures. Nucleic Acids Res.

[7] Bartonicek N, Clark MB, Quek XC, Torpy JR, Pritchard AL, Maag JLV (2017). Intergenic disease-associated regions are abundant in novel transcripts. Genome Biol.

[8] Mercer TR, Gerhardt DJ, Dinger ME, Crawford J, Trapnell C, Jeddeloh JA (2011). Targeted RNA sequencing reveals the deep complexity of the human transcriptome. Nat Biotechnol.

[9] Fachal L, Aschard A, Beesley J, Barnes DR, Allen J, Kar S, et al. Fine mapping of 150 breast cancer risk regions identifies 191 likely target genes. Nat Genet. In Press. 10.1038/s41588-019-0537-1 PMC6974400 31911677

[10] Beesley J, Sivakumaran H, Moradi Marjaneh M, Lima LG, Hillman KM, Kaufmann S, et al. Chromatin interactome mapping identifies candidate target genes at 139 independent breast cancer risk signals. Genome Biol. 2019. 10.1186/s13059-019-1877-y PMC6947858 31910858

[11] Harrow J, Frankish A, Gonzalez JM, Tapanari E, Diekhans M, Kokocinski F (2012). GENCODE: the reference human genome annotation for The ENCODE Project. Genome Res.

[12] Hon CC, Ramilowski JA, Harshbarger J, Bertin N, Rackham OJ, Gough J (2017). An atlas of human long non-coding RNAs with accurate 5′ ends. Nature..

[13] Zhao Y, Li H, Fang S, Kang Y, Wu W, Hao Y (2016). NONCODE 2016: an informative and valuable data source of long non-coding RNAs. Nucleic Acids Res.

[14] Kang YJ, Yang DC, Kong L, Hou M, Meng YQ, Wei L (2017). CPC2: a fast and accurate coding potential calculator based on sequence intrinsic features. Nucleic Acids Res.

[15] Weinstein JN, Collisson EA, Mills GB, Shaw KR, Ozenberger BA, Cancer Genome Atlas Research (2013). The Cancer Genome Atlas Pan-Cancer analysis project. Nat Genet.

[16] Andersson R, Gebhard C, Miguel-Escalada I, Hoof I, Bornholdt J, Boyd M (2014). An atlas of active enhancers across human cell types and tissues. Nature..

[17] Michailidou K, Lindstrom S, Dennis J, Beesley J, Hui S, Kar S (2017). Association analysis identifies 65 new breast cancer risk loci. Nature..

[18] Betts JA, Moradi Marjaneh M, Al-Ejeh F, Lim YC, Shi W, Sivakumaran H (2017). Long noncoding RNAs CUPID1 and CUPID2 mediate breast cancer risk at 11q13 by modulating the response to DNA damage. Am J Hum Genet.

[19] Guo H, Ahmed M, Zhang F, Yao CQ, Li S, Liang Y (2016). Modulation of long noncoding RNAs by risk SNPs underlying genetic predispositions to prostate cancer. Nat Genet.

[20] Zhang Y, He Q, Hu Z, Feng Y, Fan L, Tang Z (2016). Long noncoding RNA LINP1 regulates repair of DNA double-strand breaks in triple-negative breast cancer. Nat Struct Mol Biol.

[21] Castellanos-Rubio A, Fernandez-Jimenez N, Kratchmarov R, Luo X, Bhagat G, Green PH (2016). A long noncoding RNA associated with susceptibility to celiac disease. Science..

[22] Nik-Zainal S, Davies H, Staaf J, Ramakrishna M, Glodzik D, Zou X (2016). Landscape of somatic mutations in 560 breast cancer whole-genome sequences. Nature..

[23] De Santa F, Barozzi I, Mietton F, Ghisletti S, Polletti S, Tusi BK (2010). A large fraction of extragenic RNA pol II transcription sites overlap enhancers. PLoS Biol.

[24] Vucicevic D, Corradin O, Ntini E, Scacheri PC, Orom UA (2015). Long ncRNA expression associates with tissue-specific enhancers. Cell Cycle.

[25] Orom UA, Derrien T, Beringer M, Gumireddy K, Gardini A, Bussotti G (2010). Long noncoding RNAs with enhancer-like function in human cells. Cell..

[26] Lai F, Orom UA, Cesaroni M, Beringer M, Taatjes DJ, Blobel GA (2013). Activating RNAs associate with Mediator to enhance chromatin architecture and transcription. Nature..

[27] Xiang JF, Yin QF, Chen T, Zhang Y, Zhang XO, Wu Z (2014). Human colorectal cancer-specific CCAT1-L lncRNA regulates long-range chromatin interactions at the MYC locus. Cell Res.

[28] Gui X, Li H, Li T, Pu H, Lu D (2015). Long noncoding RNA CUDR regulates HULC and beta-catenin to govern human liver stem cell malignant differentiation. Mol Ther.

[29] Grabherr MG, Haas BJ, Yassour M, Levin JZ, Thompson DA, Amit I (2011). Full-length transcriptome assembly from RNA-Seq data without a reference genome. Nat Biotechnol.

[30] Wu TD, Watanabe CK (2005). GMAP: a genomic mapping and alignment program for mRNA and EST sequences. Bioinformatics..

[31] Trapnell C, Williams BA, Pertea G, Mortazavi A, Kwan G, van Baren MJ (2010). Transcript assembly and quantification by RNA-Seq reveals unannotated transcripts and isoform switching during cell differentiation. Nat Biotechnol.

[32] Dobin A, Davis CA, Schlesinger F, Drenkow J, Zaleski C, Jha S (2013). STAR: ultrafast universal RNA-seq aligner. Bioinformatics..

[33] Li B, Dewey CN (2011). RSEM: accurate transcript quantification from RNA-Seq data with or without a reference genome. BMC Bioinformatics.

[34] Robinson MD, McCarthy DJ, Smyth GK (2010). edgeR: a Bioconductor package for differential expression analysis of digital gene expression data. Bioinformatics..

[35] Mermel CH, Schumacher SE, Hill B, Meyerson ML, Beroukhim R, Getz G (2011). GISTIC2.0 facilitates sensitive and confident localization of the targets of focal somatic copy-number alteration in human cancers. Genome Biol.

[36] Shabalin AA (2012). Matrix eQTL: ultra fast eQTL analysis via large matrix operations. Bioinformatics..

[37] Johnston RL, Wockner L, McCart Reed AE, Wiegmans A, Chenevix-Trench G, Khanna KK (2016). High content screening application for cell-type specific behaviour in heterogeneous primary breast epithelial subpopulations. Breast Cancer Res.

[38] Yanai I, Benjamin H, Shmoish M, Chalifa-Caspi V, Shklar M, Ophir R (2005). Genome-wide midrange transcription profiles reveal expression level relationships in human tissue specification. Bioinformatics..

[39] Li Q, Seo JH, Stranger B, McKenna A, Pe'er I, Laframboise T (2013). Integrative eQTL-based analyses reveal the biology of breast cancer risk loci. Cell..

[40] Liu B, Gloudemans MJ, Rao AS, Ingelsson E, Montgomery SB (2019). Abundant associations with gene expression complicate GWAS follow-up. Nat Genet.

