# Supplementary material for: Non-coding RNAs underlie genetic predisposition to breast cancer
Source: Genome Biol. 2020 Jan 7;21:7. doi: 10.1186/s13059-019-1876-z (PMC6947989; doi:10.1186/s13059-019-1876-z)
Supplement: Supplementary file 1 — Additional file 1: Figure S1. RNA CaptureSeq performance. Figure S2. Quality control and properties of captured transcripts. Figure S3. Validation of captured transcripts. Figure S4. Co-localisation of eQTL associations at breast cancer GWAS signals. Figure S5. eQTL analysis and chromatin interactions at breast cancer risk regions. [file 13059_2019_1876_MOESM1_ESM.docx]

**Supplementary Figures**

Fig. S1


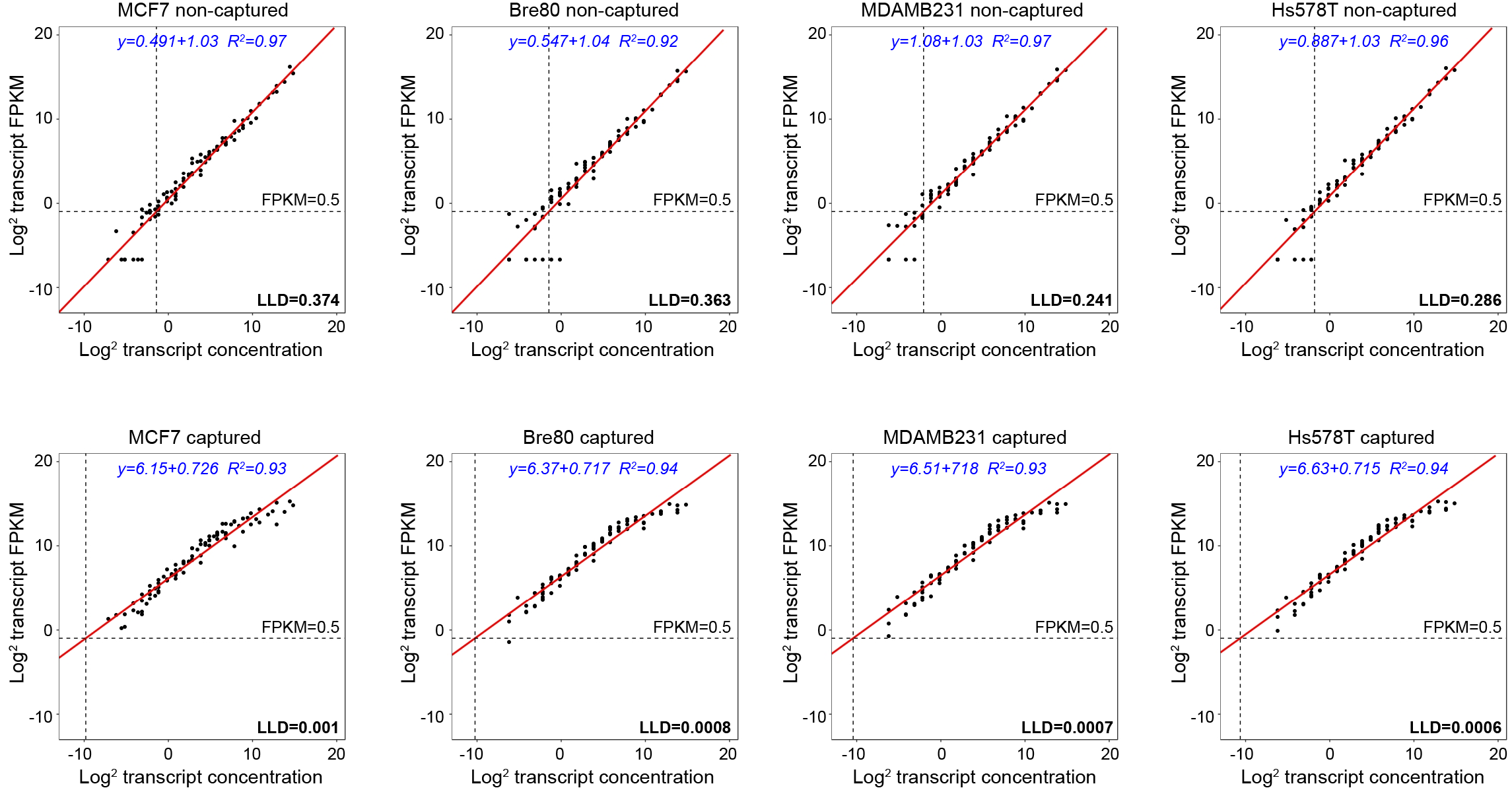


**RNA CaptureSeq performance.** Assessment of RNA CaptureSeq sensitivity using ERCC spike-in transcripts. A dose-response curve was generated by plotting normalised expression (log2 (FPKM)) of the ERCC spike-in transcripts against their known molar concentrations (log2(attomoles/ul)) for each library (top panels, non-captured; bottom panels, captured). The best-fit line for each dose-response curve was plotted by linear regression and used to identify lower limit of detection (LLD, the molar concentration value where the best-fit line crosses the sensitivity threshold of 0.5 FPKM).

Fig. S2


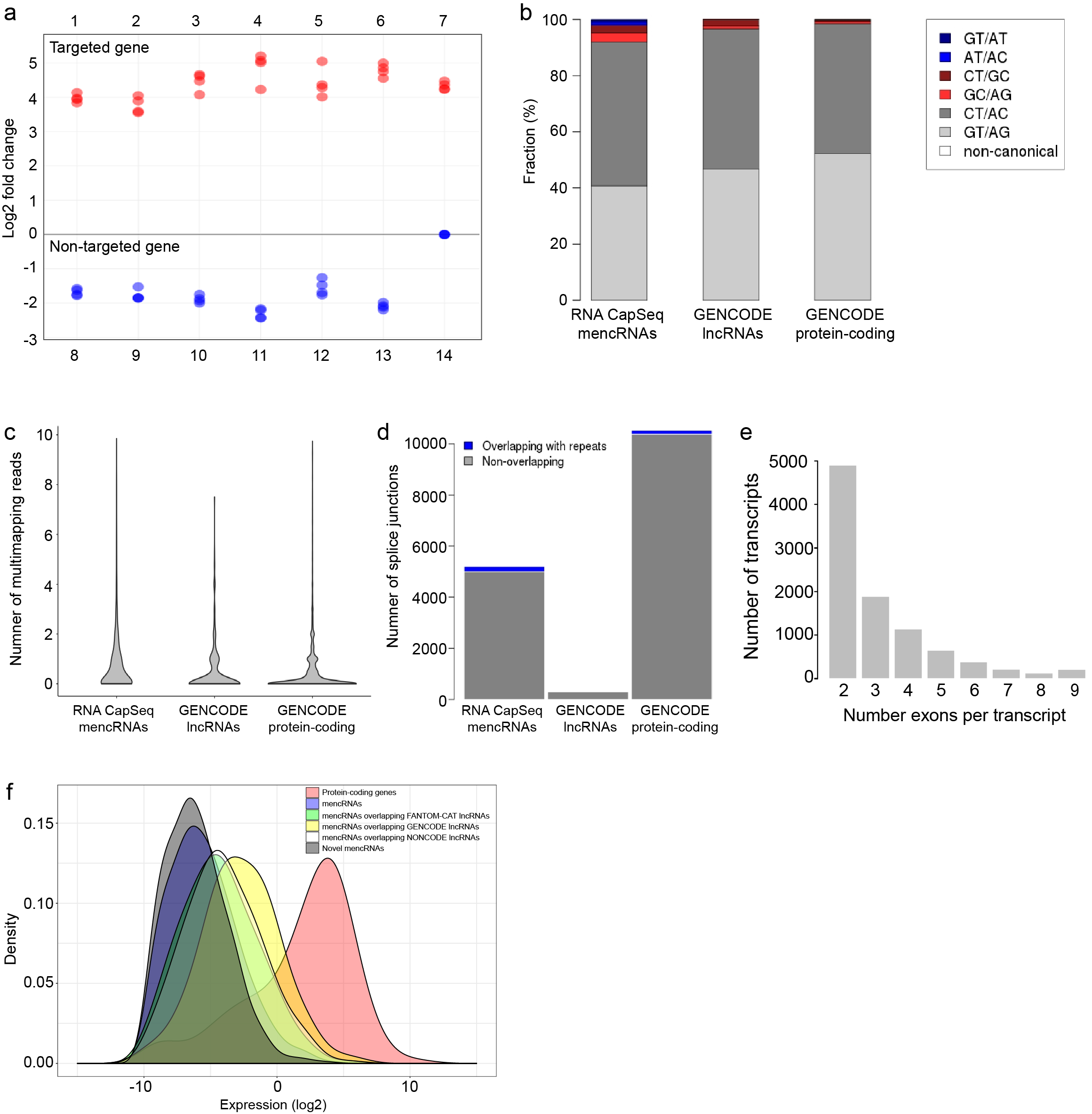


**Quality control and properties of captured transcripts. a** Assessment of RNA CaptureSeq specificity using seven control genes. The expression fold change (captured vs. non-captured libraries; y-axis) of targeted (red dots) and non-targeted (blue dots) genes are shown. Seven housekeeping genes were targeted in the RNA CaptureSeq (1-7; *GUSB, HMBS, HPRT1, NLK, RUNX2, TBP, TFRC,* respectively). For each housekeeping gene, the nearest protein-coding gene not included in the design is plotted as “non-targeted” (8-14; *VKORC1L1, H2AFX, PHF6, TMEM97, SUPT3H, PSMB1* and *ZDHHC19*, respectively). Types of splice junctions **b**, number of assembled transcripts overlapping with repeat sequences **c** and number of multimapping reads crossing the splice junctions **d** in RNA CaptureSeq identified mencRNAs, annotated GENCODE lncRNAs and annotated GENCODE protein-coding genes. **e** Number of exons per mencRNA transcript. Pooled captured transcripts from all libraries were binned based on their exon numbers. **f** Expression distribution of mencRNAs versus annotated lncRNAs and protein-coding genes. MencRNAs with max. FPKM ≥0.5 were mapped in TCGA RNA-Seq data. Average expression across the TCGA tumors were compared between total mencRNAs (n = 4020), novel mencRNAs (n = 2766), those overlapping GENCODE (n = 328), FANTOM-CAT (n = 782) and NONCODE lncRNAs (n = 1016) and protein-coding genes (n = 1087). The y-axis represents the frequency of transcripts with a given expression value represented as log2 (average FPKM) on the x-axis.

Fig. S3


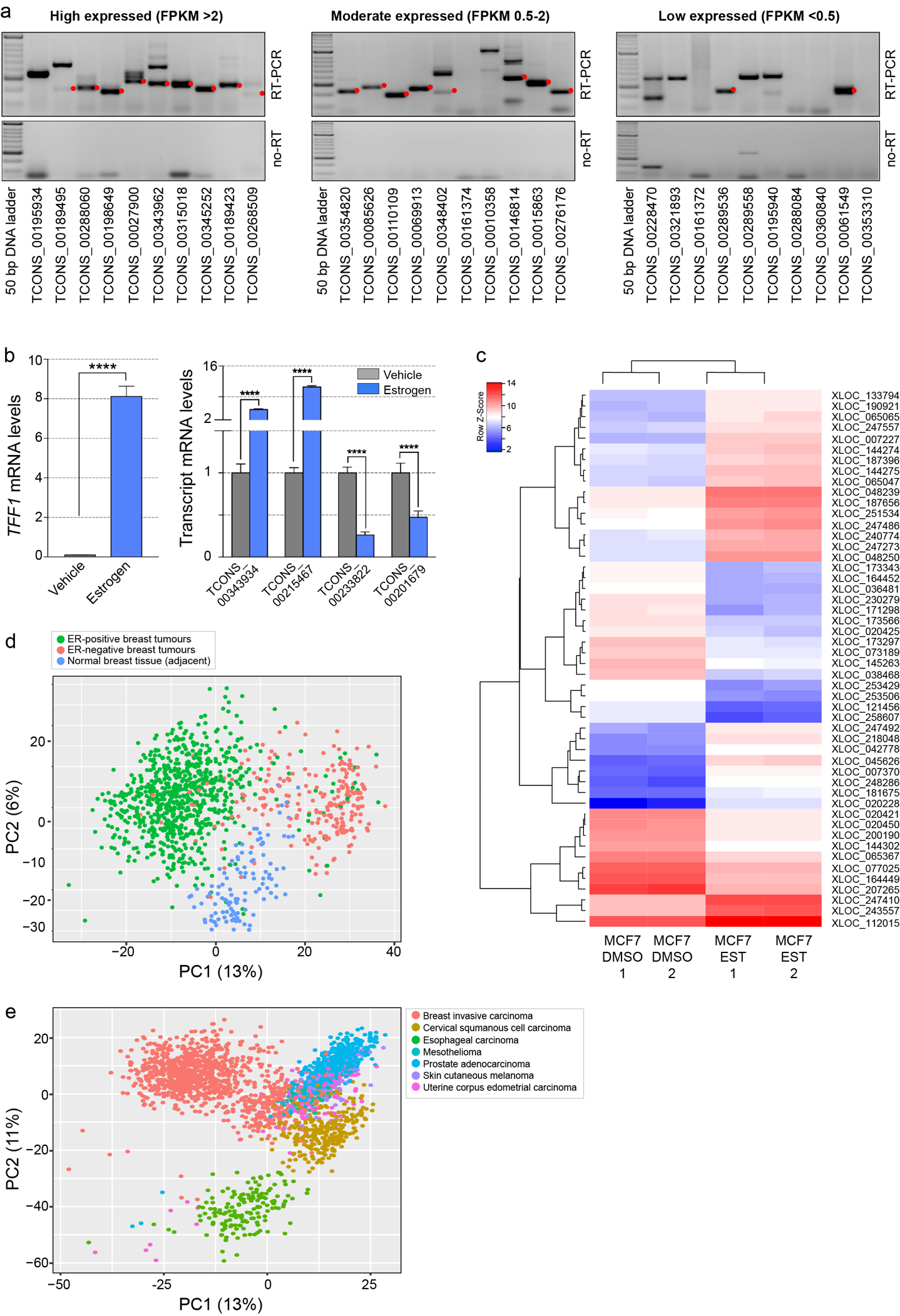


**Validation of captured transcripts. a** RT-PCR validation of CCV-containing captured transcripts binned into three groups based on their level of expression. Primers are listed in **Additional file 2:** **Table S15.** **b** TaqMan qPCR confirming estrogen induction in MCF7 cells. Left panel: *TFF1* mRNA normalised to *GUS* mRNA as an internal control. Right panel: qPCR of representative estrogen-induced (TCONS-00343937, TCONS-00215467) and estrogen-repressed (TCONS-00343937, TCONS-00215467) captured transcripts. Error bars denote SEM (N=3). *P* values were determined with a two-tailed t test (****p < 0.0001). **c** Heatmap of top 50 differentially expressed genes between MCF7 estrogen-treated and control libraries. Gene expressions (log2CPM (counts-per-million)) are represented as color gradients. Principal component analysis (PCA) of captured transcripts in **d** ER-positive breast tumors, ER-negative breast tumors and normal adjacent breast tissue from TCGA and **e** seven different tumor tissues from TCGA. Scaled, centred, and normalised expression of the captured transcripts were analysed for the first (x-axis; PC1) and second (y-axis; PC2) principal components. Each dot represents expression profile of an individual sample.

Fig. S4


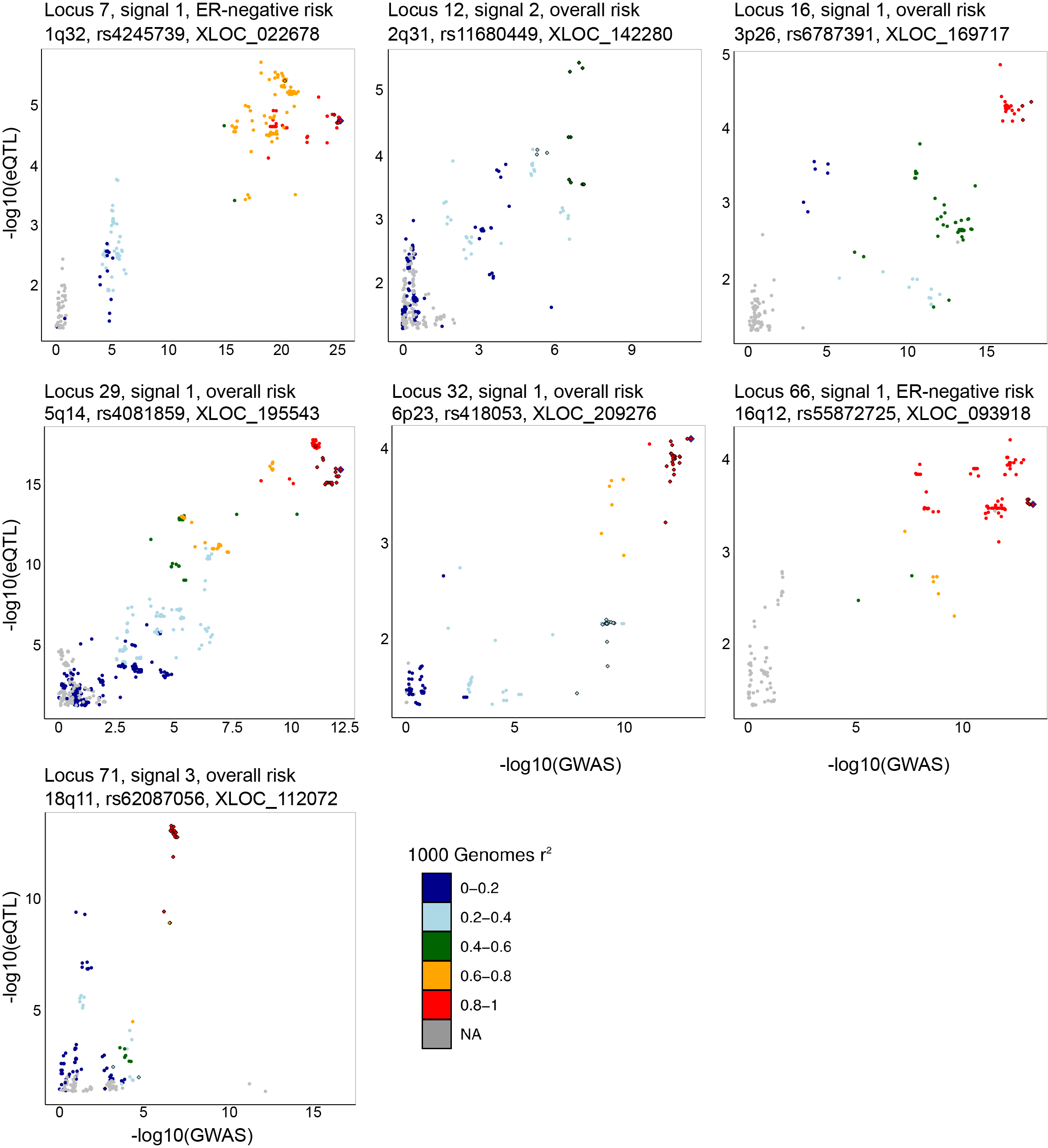


**Co-localisation of eQTL associations at breast cancer GWAS signals.** SNPs are plotted if they are significant for association with expression (*P* < 0.05). Each SNP is coloured based on LD pairwise correlation (1000 Genomes EUR population) with the top CCV, which are shown as large purple diamonds if also present in the association data. Other CCVs are represented as colored diamonds. Note that at locus 12, signal 2 (2q31, rs11680449, *XLOC-142280*), an eQTL signal for the strongest CCVs was not detected, because not all the CCVs could be confidently imputed using TCGA Affemetrix v6.0 chips.

Fig. S5


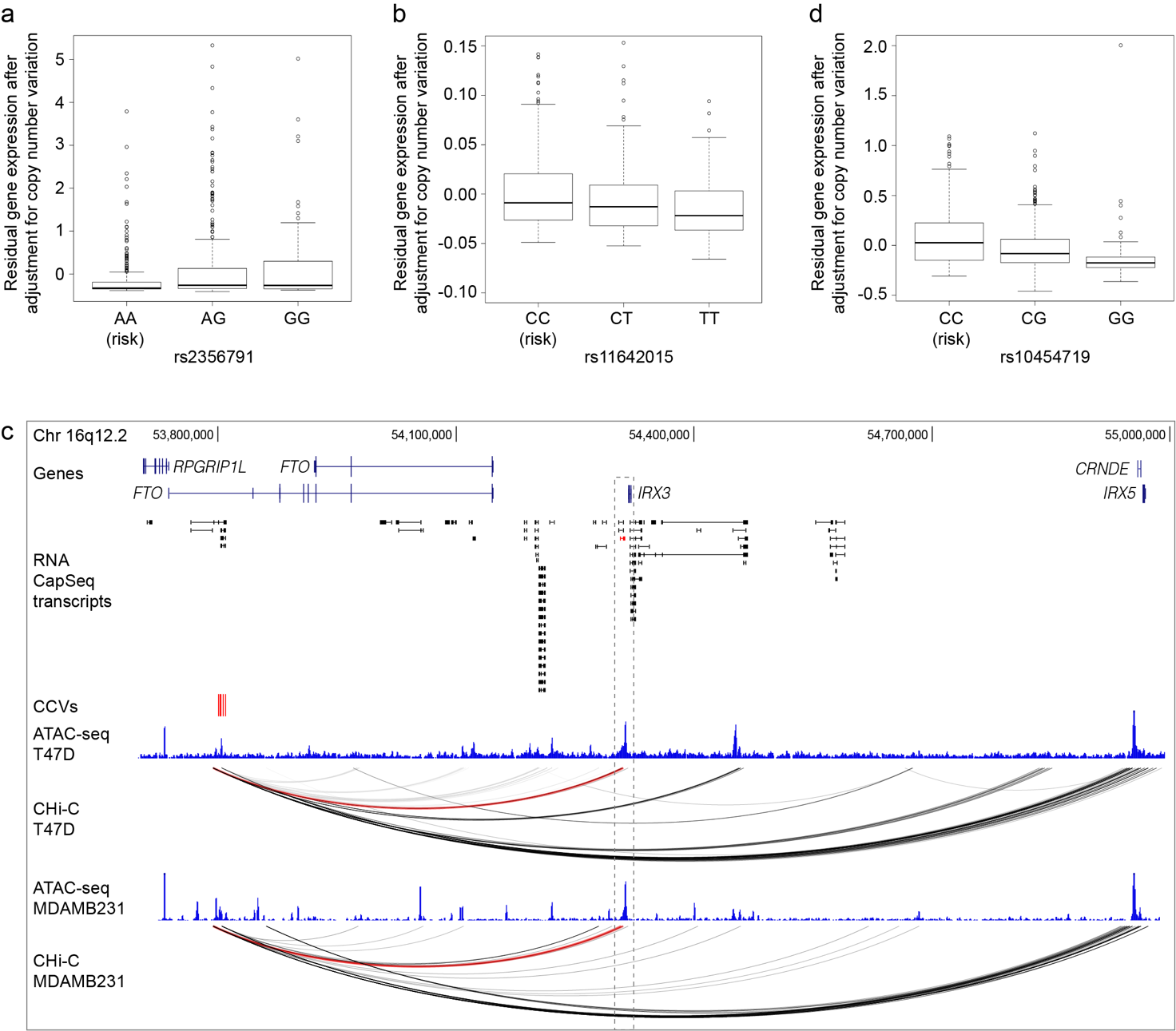


**eQTL analysis and chromatin interactions at breast cancer risk regions.** **a,b,d** eQTL analysis and association of CCVs with mencRNA expression in the TCGA dataset. The *x*-axis of each plot corresponds to the three observed SNP genotypes and the *y*-axis represents log2-normalized gene expression values. **c** WashU genome browser showing annotated GENCODE genes (blue) and mencRNAs (black). The *XLOC-93918* mencRNA is highlighted in red. CCVs are shown as red vertical lines. The ATAC-seq data are shown as dark blue histograms and CHi-C identified chromatin interactions are shown as arcs from T47D and MDAMB231 breast cancer cell lines. Red arcs depict chromatin looping between CCVs and the *XLOC-93918* promoter region. The dashed grey outline highlights the *XLOC-93918* mencRNA, ATAC-seq peaks and relevant chromatin interactions.
