# Supplementary material for: Non-coding RNAs underlie genetic predisposition to breast cancer
Source: Genome Biol. 2020 Jan 7;21:7. doi: 10.1186/s13059-019-1876-z (PMC6947989; doi:10.1186/s13059-019-1876-z)
Supplement: Supplementary file 3 — Additional file 3. Review history. [file 13059_2019_1876_MOESM3_ESM.docx]

**Review history**

**1^st^ Round**

**Reviewer 1**

Overall, I want to congratulate the authors for their efforts and tons of genome-wide expression data and own expression data and qRT-PCR studies, providing an interesting and novel picture of variants and breast cancer risk alleles in non-coding RNA areas.
Though this part of the manuscript is strong and interesting with novelity, the authors lack to show (at least some) examples of the functional role of some of their non-coding RNA molecules involved.
Knock-in and knock-out studies for risk alleles, phenotype changes in cell lines, GMOs models and some mechnistic insights for the most promising candidates would be helpful in order to assess to value of the many new discoveries and hypothesis suggested by the authors.

**Reviewer 2**

The study by Marjaneh and colleagues applies RNA-capture sequencing to breast cancer cells to uncover lncRNAs overlapping GWAS hits. The demonstrate that exons of these lncRNAs are enriched for credible causal variants and highlight a few cases where CCVs interact with lncRNA promoters through chromatin looping. Overall, I believe this is a very elegant study that once more highlights the association of GWAS hits with expressed lncRNAs. The results from this study are definitely relevant to support ongoing efforts in trying to understand the functional implication of GWAS loci. Nevertheless, I feel the study should provide more details on various aspects that are discussed. 

The authors mention a 140-fold lower expression between mencRNAs and protein coding genes. This seems higher than what is classically reported for lncRNAs. What is the average expression difference between their mencRNAs and all gencode lncRNAs?

TCGA RNA-seq data is unstranded. Has this been taken into account when assessing mencRNA expression levels in TCGA data and correlation with protein coding gene expression? 

What is the fraction of mencRNAs that have CAGE peaks of H3K4me3/H3K27ac peaks overlapping their TSS? Are all mencRNAs independently transcribed?

How specific are these mencRNAs for breast cancer compared to other cancer tissues? Did the authors evaluate expression levels in other TCGA cancer types?

The enrichment of CCVs is highest in protein coding gene introns. Are these introns preferentially overlapping with mencRNA exons?

The 16q12.2 CCVs also contact the promoter/first intron of the FTO gene. Was eQTL detected for FTO?

Could CCVs in mencRNA exons for which no eQTL was observed modulate structure of the mencRNAs? Are these sites more highly conserved than other exons?

In Figure 4, it could be helpful to also include H3K4me1, H3K4me3 and HRK27ac tracks. Is there for instance evidence for enhancer activity at the IRX5 and/or FTO loci involved in the CCV looping? Also in related figures, these tracks could be helpful. 

Did the capture probes in the 6q25 locus also capture ESR1? Does the RNA-capture seq data validate the observed correlations between ESR1 and the two mencRNAs? 

The authors claim that in the presence of looping, expression correlation between mencRNA-mRNA pairs increases. While there is a statistically significant difference, the effect size is marginal. I would suggest to at least include this critical note in the description of this result.

It would be really helpful to put together an overview figure demonstrating what fraction of CCVs is associated to mencRNAs, protein-coding genes, regulatory elements etc. How much of the unannotated GWAS hits could be linked to mencRNAs? How much of these show signs of eQTL?

**Reviewer 3**

Marjaneh et al evaluated the potential effect of non-coding RNAs (multi-exonic non-coding RNAs or mencRNAs) as mediators of common variant associations with breast cancer risk. The paper includes novel data that identifies mencRNAs (based on targeted capture of regions in cis) in benign breast tissue, primary breast tumor samples and breast cell lines. In addition, analyses of TCGA are also pursued. The authors conclude that at several loci, breast cancer SNPs may act via alteration of the sequence of expressed non-coding RNAs or via control of overall mencRNA expression. The paper makes a potentially important contribution to the possible mechanisms underlying GWAS hits, and is therefore of interest not only to geneticists studying breast cancer, but also to geneticists and genome biologists studying complex traits and following up on GWAS loci from many other diseases. 

However, there are some issues with the analyses and the presentation of results as noted below:

1. The analysis of enrichment of expressed regions (Fig 1) has several issues. If I understand this correctly, the reviewers asked the question of whether CCV overlap was significantly enriched for each annotation. The P values presented are from permutation of the annotation. But the paper states that only protein-coding and non-coding RNA annotations were used to generate the "null". Why weren't all of the different annotations permuted? The choice of protein coding regions to generate the null includes a major potential confounder, that of allele frequency and LD. Protein coding variants are systematically lower in number of variants and in allele frequency of those variants. Thus, protein coding are likely to be under-represented in any enrichment test for any common variant set. Generating the correct null is always a challenge with these types of analyses since LD and allele frequency are such important parameters in determining potential CCV overlap. Also the way the data are presented in Fig 2a makes it more difficult to understand the results since Z statistics do not give the reader a sense of absolute numbers involved and fold-enrichment. What is needed is both the numerator and denominator for (ie # of SNPs total) for each of the annotations, preferably stratified by MAF. 

Another way to do this that may get around the issue of LD is to look at heritability enrichment within these results adjusting for LD using LD score regression (see Finucane et al Nat Genetics 2018) 

2. The eQTL analyses are a rational alternative mechanism to pursue (eg mencRNA enhancers). However, a major concern is that eQTLs in cis may coincidentally overlap the breast cancer association signals. To address this, the authors can do colocalization analyses (e.g. COLOC package in R or similar type of approach) to evaluate whether, after accounting for LD at the region, the CCV for the eQTL signal are overlapping or mutually exclusive with the CCV for breast cancer association. 

3. The argument that exonic non-coding variants affect stability seems weak, since there are 119 mencRNAs in 69/134 breast cancer signals but only 10 of the mencRNAs (of 119?) are eQTLs and of these, 6/10 are in exons. So if interpreted correctly, only 4/119 mencRNAs which have exonic variants which overlap with CCV. Thus, the vast majority of CCV in exons do not seem to affectd expression. In this case, is the overlap coincidental? Or are other mechanisms plausible? If so, what are they?

4. The analysis of AEI uses a p value of 0.05 which is not adjusted for multiple hypothesis testing and thus likely includes lots of false positives. FDR should be used for this analysis (as it was used in other analyses). Also the methods for the AEI section state that 200 samples from TCGA and GTEX were used. However, the numbers of samples in figure 5a are <<200 (12, 17 and 11). Why are the numbers in Fig 5a so small? The numbers of heterozygotes for these SNPs should be much higher. Also data from TCGA should have far more samples (>800 samples with RNA-seq are available). Finally the methods state that GTEX and TCGA were used but the figure states that TCGA is used. Please clarify which is true. If both GTEX and TCGA are used, that should be avoided since gene expression is likely to be different between tumor and normal and since genotypes are also systematically different between TCGA and normal (as these are breast cancer risk SNPs). 

5. There is no summary of the oveall evidence in terms of loci and/or non-coding RNAs. For the sake of presentation, it would be helpful to have a summary of the proposed non-coding RNA-SNP pairs proposed by the authors as potential mediators of the GWAS signals, and what the evidence is for each of them (eg: within exon, eQTL, AEI, etc.) This would also help to determine whether there is concordant evidence for any of these analyses.

6. The discussion should try to summarize to what degree the evidence is consistent or not for each type of analysis for each of the SNP-mencRNA pairs proposed. For example, for eQTLs there should generally be some evidence of

**Response to reviewers**

**Reviewer #1**

Overall, I want to congratulate the authors for their efforts and tons of genome-wide expression data

and own expression data and qRT-PCR studies, providing an interesting and novel picture of

variants and breast cancer risk alleles in non-coding RNA areas. Though this part of the manuscript

is strong and interesting with novelity, the authors lack to show (at least some) examples of the

functional role of some of their non-coding RNA molecules involved. Knock-in and knock-out studies

for risk alleles, phenotype changes in cell lines, GMOs models and some mechanistic insights for

the most promising candidates would be helpful in order to assess to value of the many new

discoveries and hypothesis suggested by the authors.

We thank the reviewer for their positive comments. However, additional functional studies for

individual mencRNAs is a substantial amount of work and outside the scope of the current study.

**Reviewer #2**

The study by Marjaneh and colleagues applies RNA-capture sequencing to breast cancer cells to

uncover lncRNAs overlapping GWAS hits. The demonstrate that exons of these lncRNAs are

enriched for credible causal variants and highlight a few cases where CCVs interact with lncRNA

promoters through chromatin looping. Overall, I believe this is a very elegant study that once more

highlights the association of GWAS hits with expressed lncRNAs. The results from this study are

definitely relevant to support ongoing efforts in trying to understand the functional implication of

GWAS loci. Nevertheless, I feel the study should provide more details on various aspects that are

discussed.

1. The authors mention a 140-fold lower expression between mencRNAs and protein coding genes.

This seems higher than what is classically reported for lncRNAs. What is the average expression

difference between their mencRNAs and all gencode lncRNAs?

We have now compared the expression of the mencRNAs (n = 4020) and GENCODE lncRNAs

detected by our RNA CaptureSeq (n = 328). On average, the mencRNAs show ~5.5 times lower

expression. This is likely due to our targeted RNA sequencing approach, which enabled identification

of transcripts with lower expression levels. This data is included as a new **Figure S2f** and a comment

made in the text (Pages 6-7).

2. TCGA RNA-seq data is unstranded. Has this been taken into account when assessing mencRNA

expression levels in TCGA data and correlation with protein coding gene expression?

The majority of identified mencRNAs are either intergenic (44.4%) or intronic (51.0%) to proteincoding

genes. For the intronic mencRNAs, the RNAseq reads coming from pre-mRNAs would be

almost negligible therefore we have kept these in our TCGA expression analyses. However, we have

now removed the 4.6% of mencRNAs which overlap (>1bp) with exons of protein-coding genes.

Please note that the PCA figures (**Figs. 1f,g** and **S3d,e**) have now been updated. In addition, one of

the eQTLs which colocalized with a breast cancer signal also overlapped with exons of a proteincoding

gene (XLOC-019547). We have now removed this eQTL and updated the results and **Table**

**S11** accordingly.

3. What is the fraction of mencRNAs that have CAGE peaks of H3K4me3/H3K27ac peaks

overlapping their TSS? Are all mencRNAs independently transcribed?

CAGE-seq and H3K4Me3/HeK27Ac ChIP-seq data was only available for MCF7 and HMEC cells.

Approximately 24% of mencRNA transcripts expressed in MCF7 cells (FPKM ≥ 0.5) have evidence

for promoter activity as based on CAGE, H3K4Me3 or H3K27Ac peaks. There are many reasons for

this low number including: 1) The expression of many mencRNAs are low therefore CAGE,

H3K4Me3 and H3K27Ac peaks may not be detectable. 2) It is not possible to assign expression to

individual mencRNA transcripts when other isoforms share mencRNA exons (as mapping of the

sequencing reads cannot distinguish between isoforms). This is particularly difficult for lncRNAs as

compared to protein-coding genes as lncRNAs generally have more alternative splice isoforms and

multiple transcription start sites (PMID: 29396323). 3) The MCF7 cell line is notoriously different

between labs (PMID: 300899904) therefore the expression of mencRNAs in the MCF7 line in our lab

may be different to that used by the ENCODE and FANTOM Consortia from which the histone

modification CHIP-seq and CAGE-seq was performed. For these reasons, we have not included

these datasets in the manuscript.

4. How specific are these mencRNAs for breast cancer compared to other cancer tissues? Did the

authors evaluate expression levels in other TCGA cancer types?

We thank the reviewer for this comment and have now evaluated the expression of our mencRNAs

in six other cancer types from TCGA. We show that 79.9% of mencRNAs exhibit high tissue

specificity (tissue specificity index (Tau) of ≥ 0.5) as compared to 31.6% for protein-coding genes

(Page 7 and new **Fig. 1h**). Furthermore, PCA analyses show that the different tumor types tightly

cluster based on mencRNA expression (new **Fig. S3e**).

5. The enrichment of CCVs is highest in protein coding gene introns. Are these introns preferentially

overlapping with mencRNA exons?

Within the captured regions, the total number of genomic intervals corresponding to GENCODE v19

protein-coding introns is 10,809. The subset (n = 10,480) of protein-coding introns which do not

contain CCVs include 1,508 which overlap mencRNA exons (14%). Of the subset which do contain

CCVs (n=329), 99 contain mencRNA exons (30%). This represents a >2-fold enrichment (*P* = 8.2e-

13, Fishers Exact test). We don’t think this adds much to the current manuscript therefore this has

not been included.

6. The 16q12.2 CCVs also contact the promoter/first intron of the FTO gene. Was eQTL detected

for FTO?

The chromatin interaction from the CCVs is to the 3’ end of FTO (**Fig. 4b**). However, no eQTL was

detected for FTO or any other gene in the region (PMID: 29059683). We have now clarified this in

the text (Page 10).

7. Could CCVs in mencRNA exons for which no eQTL was observed modulate structure of the

mencRNAs? Are these sites more highly conserved than other exons?

Yes. As discussed in the manuscript (Discussion, paragraph 3), we believe it is plausible that CCVs

may alter the structure of mencRNAs. As suggested by the reviewer, we assessed conservation of

mencRNA exons using *UCSC phastCons100way* and surprisingly observed less conservation

scores for the mencRNA exons overlapping with CCVs compared to those not overlapping with the

CCVs. This could be explained by potential detrimental effects of the CCVs on the mencRNAs.

However, we believe this is too speculative so have not included this in the manuscript.

8. In Figure 4, it could be helpful to also include H3K4me1, H3K4me3 and HRK27ac tracks. Is there

for instance evidence for enhancer activity at the IRX5 and/or FTO loci involved in the CCV looping?

Also in related figures, these tracks could be helpful.

We have now included available tracks in **Figs. 4, 5 and 6**. Consistent with enhancer activity, both

H3K4me1 and H3K27ac peaks, but not H3K4me3 are found in this region. We have now clarified

this in the text (Page 10).

9. Did the capture probes in the 6q25 locus also capture ESR1? Does the RNA-capture seq data

validate the observed correlations between ESR1 and the two mencRNAs?

No. We deliberately did not capture protein-coding exons at any risk region, therefore this analysis

is not possible.

10. The authors claim that in the presence of looping, expression correlation between mencRNAmRNA

pairs increases. While there is a statistically significant difference, the effect size is marginal.

I would suggest to at least include this critical note in the description of this result.

This has now been noted in the manuscript (Page 12).

11. It would be really helpful to put together an overview figure demonstrating what fraction of CCVs

is associated to mencRNAs, protein-coding genes, regulatory elements etc. How much of the

unannotated GWAS hits could be linked to mencRNAs? How much of these show signs of eQTL?

We understand where the reviewer is coming from, however as some of the signals show evidence

for both protein-coding and mencRNAs being involved it was too difficult to depict in a figure. We

have therefore included this information in a new **Table S11 (Page 8)**.

**Reviewer #3**

Marjaneh et al evaluated the potential effect of non-coding RNAs (multi-exonic non-coding RNAs or

mencRNAs) as mediators of common variant associations with breast cancer risk. The paper

includes novel data that identifies mencRNAs (based on targeted capture of regions in cis) in benign

breast tissue, primary breast tumor samples and breast cell lines. In addition, analyses of TCGA are

also pursued. The authors conclude that at several loci, breast cancer SNPs may act via alteration

of the sequence of expressed non-coding RNAs or via control of overall mencRNA expression. The

paper makes a potentially important contribution to the possible mechanisms underlying GWAS hits,

and is therefore of interest not only to geneticists studying breast cancer, but also to geneticists and

genome biologists studying complex traits and following up on GWAS loci from many other

diseases.

However, there are some issues with the analyses and the presentation of results as noted below:

1. The analysis of enrichment of expressed regions (Fig 1) has several issues. If I understand this

correctly, the reviewers asked the question of whether CCV overlap was significantly enriched for

each annotation. The P values presented are from permutation of the annotation. But the paper

states that only protein-coding and non-coding RNA annotations were used to generate the "null".

Why weren't all of the different annotations permuted?

This may be a misunderstanding, each of the annotations (exon, intron, or promoter) for both proteincoding

or non-coding genes were permuted separately to generate a null background set of intervals.

These separate background sets were then compared to the observed overlap between CCVs and

that specific annotation. We have now edited the methods section to clarify this point (Page 21).

The choice of protein coding regions to generate the null includes a major potential confounder, that

of allele frequency and LD. Protein coding variants are systematically lower in number of variants

and in allele frequency of those variants. Thus, protein coding are likely to be under-represented in

any enrichment test for any common variant set. Generating the correct null is always a challenge

with these types of analyses since LD and allele frequency are such important parameters in

determining potential CCV overlap.

We agree with the reviewer that these characteristics make it difficult to accurately model a null

distribution for SNP overlap analyses. The method presented in the paper relies on a random

background generated by moving the exons within the captured regions while keeping the positions

of the SNPs constant. This method is therefore less sensitive to biases encountered for SNP

matching methods (PMID: 26140449).

Also the way the data are presented in Fig 2a makes it more difficult to understand the results since

Z statistics do not give the reader a sense of absolute numbers involved and fold-enrichment. What

is needed is both the numerator and denominator for (ie # of SNPs total) for each of the annotations,

preferably stratified by MAF.

We agree that interpretation of Z scores is difficult in the context of number of overlapping events.

We now present the results as bar plots depicting the absolute number of observed and expected

CCV intersections based on random permutations (new **Fig. 2a**). The number of CCVs which overlap

each annotation is now also presented in a new **Table S10**.

Another way to do this that may get around the issue of LD is to look at heritability enrichment within

these results adjusting for LD using LD score regression (see Finucane et al Nat Genetics 2018)

We agree with the reviewer that LD score regression methods are likely to circumvent many of the

problems mentioned above. Unfortunately, we are impeded on two fronts: 1) the design of the

genotyping expression (the OncoArray) relied on densely typing SNPs at specific loci found

previously through GWAS; and 2) the targeted design of the RNA CaptureSeq experiment which

included only a subset of the genome-wide significant loci. As both aspects were highly selected,

the introduced bias would render results difficult to interpret. In support of this, the LD score

regression wiki notes that *“LD Score regression doesn’t work well with targeted genotyping arrays”.*

2. The eQTL analyses are a rational alternative mechanism to pursue (eg mencRNA enhancers).

However, a major concern is that eQTLs in cis may coincidentally overlap the breast cancer

association signals. To address this, the authors can do colocalization analyses (e.g. COLOC

package in R or similar type of approach) to evaluate whether, after accounting for LD at the region,

the CCV for the eQTL signal are overlapping or mutually exclusive with the CCV for breast cancer

association.

We considered eQTLs to “overlap” with breast cancer risk signals based on the *P*-value for the top

eQTL SNP being within two orders of magnitude of the eQTL *P-*value for a CCV. However, we have

now made this clearer in the results section (Page 9). Furthermore, we have now examined the

colocalization of the eQTL and breast cancer risk signals by plotting the P-values of the eQTLs for

each SNP in the region vs P-values of the association with breast cancer risk (as performed in PMID:

31043754). Seven of the nine eQTLs colocalize with breast cancer risk signals (new **Fig. S4** and

updated **Table S11**).

3. The argument that exonic non-coding variants affect stability seems weak, since there are 119

mencRNAs in 69/134 breast cancer signals but only 10 of the mencRNAs (of 119?) are eQTLs and

of these, 6/10 are in exons. So if interpreted correctly, only 4/119 mencRNAs which have exonic

variants which overlap with CCV. Thus, the vast majority of CCV in exons do not seem to affected

expression. In this case, is the overlap coincidental? Or are other mechanisms plausible? If so, what

are they?

There are many reasons why protein-coding or mencRNA eQTLs are not identified including: 1) The

eSNPs are acting in multiple tissues, but our CCVs only act in one specific cell type. 2) Given normal

breast tissue is so heterogeneous, any effect that is specific to one cell type (such as stem cells) is

would be significantly diluted and 3) the eQTLs may be context dependent so might only be present

in breast tissue under particular stimuli or stages of development. For example, the relevant cells for

the analysis could be luminal progenitor cells in adolescence, when the human breast is susceptible

to environmental and hormonal influences.

Of note, using the same TCGA data set, we have identified protein-coding eQTLs that overlap (based

on the *P*-value for the top eQTL SNP being within two orders of magnitude of the eQTL *P-*value for

a CCV) with only ~20% of breast cancer risk signals suggesting that there is something inherent

about breast cancer that impedes the identification of eQTLs (as suggested above).

For the mencRNA eQTLs we identified where an eQTL SNP falls in a mencRNA exon, it is not

possible to know whether the CCVs affect the stability of the mencRNAs or other mechanisms are

at play, especially since highly correlated SNPs (CCVs within the same signal) lie outside the exons

of the mencRNAs which could potentially alter the function of nearby regulatory elements of the

mencRNAs (eg. mencRNA promoters or enhancers). As shown for Figs. 4, 5 and 6, we provide

evidence that some of the eQTLs act through long range enhancers. However, additional lab based

experiments will be required to confirm these observations. We have expanded the discussion

accordingly (Page 15).

4. The analysis of AEI uses a p value of 0.05 which is not adjusted for multiple hypothesis testing

and thus likely includes lots of false positives. FDR should be used for this analysis (as it was used

in other analyses). Also the methods for the AEI section state that 200 samples from TCGA and

GTEX were used. However, the numbers of samples in figure 5a are <<200 (12, 17 and 11). Why

are the numbers in Fig 5a so small? The numbers of heterozygotes for these SNPs should be much

higher. Also data from TCGA should have far more samples (>800 samples with RNA-seq are

available). Finally the methods state that GTEX and TCGA were used but the figure states that TCGA

is used. Please clarify which is true. If both GTEX and TCGA are used, that should be avoided since

gene expression is likely to be different between tumor and normal and since genotypes are also

systematically different between TCGA and normal (as these are breast cancer risk SNPs).

The number of samples used in the original **Fig. 5a** was dependent on whether the mencRNA

expression was high enough to conduct calculate allelic imbalance. Many samples had to be

excluded due to little or no expression of the mencRNA in those individuals. After FDR correction

we did not identify significant associations between AEI of mencRNAs and CCVs (FDR < 0.05). This

likely reflects the small sample size used for the analysis, but the reviewer is correct in that there are

likely false positives, and therefore we have removed the AEI analyses from the manuscript.

While we originally identified the 6q25 mencRNAs through AEI analyses, the promoters of these

three mencRNAs also participate in chromatin interactions with regions containing CCVs and are

listed in **Tables S11 and S13**. Given the interest for ESR1 in breast cancer, we have kept this Figure

in the manuscript (without the AEI analyses) as it provides another good example of CCVs potentially

working through distal regulation of mencRNAs genes.

5. There is no summary of the overall evidence in terms of loci and/or non-coding RNAs. For the

sake of presentation, it would be helpful to have a summary of the proposed non-coding RNA-SNP

pairs proposed by the authors as potential mediators of the GWAS signals, and what the evidence

is for each of them (eg: within exon, eQTL, AEI, etc.) This would also help to determine whether

there is concordant evidence for any of these analyses.

Please see response to Reviewer 2 (Q11).

6. The discussion should try to summarize to what degree the evidence is consistent or not for each

type of analysis for each of the SNP-mencRNA pairs proposed. For example, for eQTLs there should

generally be some evidence of

It is not clear what the reviewer is asking here as the final sentence has been cut-off. However, our

new **Table S11** provides a tabulated summary of the evidence for mencRNAs involvement at breast

cancer risk signals and we now highlight this in the discussion (Page 13).

**2^nd^ Round**

**Reviewer 2**

The authors have adequately addressed the comments that were raised. I have no further comments.

**Reviewer 3**

Overall, the manuscript has improved greatly. The additional of supplementary table 11 is a great help in summarizing the results. There are minor modifications recommended to supplementary figure 4 (see below). 
Otherwise, I think this manuscript provides an important analysis of breast cancer GWAS loci.

The authors have substantially improved the manuscript. The current version makes the case more clearly that these mencRNAs could be mediating at least some of the breast cancer GWAS associations (and, by extension, may also be a mechanism for other GWAS loci.) 

My only recommendation is to make minor modifications to figure S4 would help clarify/improve that figure. First, it would help to have the actual loci and/or index SNPs used in the figure. (As it stands now, the reader has to find the loci and the RNAs in supplemental tables which is possible, but unnecessary). Second, the locus 12 figure should include the top SNPs. (The figure legend declares that the top CCVs are not eQTLs. But all of the SNPs at the locus should be included in the figure, otherwise the correlation in the figure is misleading.) Finally, please check/clarify the RNA names. I could not find XLOC_0222678 or XLOC_209276 in Suppl Table 2. Were these typos for different RNA names?
